# Habitat radiomics predicts occult lymph node metastasis and uncovers immune microenvironment of head and neck cancer

**DOI:** 10.1186/s12967-025-06474-7

**Published:** 2025-05-01

**Authors:** Xinwei Chen, Huan Jiang, Min Pan, Chengmin Feng, Yanshi Li, Lin Chen, Yuxi Luo, Long Liu, Juan Peng, Guohua Hu

**Affiliations:** 1 Department of Otorhinolaryngology, The First Affiliated Hospital of Chongqing Medical University, Chongqing, China; 2 Department of Radiology, The First Affiliated Hospital of Chongqing Medical University, Chongqing, China; 3 Department of Radiology, Zigong Fourth People’s Hospital, Zigong, China; 4 Department of Radiology, The People’s Hospital of Hechuan, Chongqing, China

**Keywords:** Head and neck squamous cell carcinoma, Radiogenomics, Habitat radiomics, Occult lymph node metastasis, Immune microenvironment

## Abstract

**Background:**

Occult lymph node metastasis (LNM) is a key prognostic factor for patients with head and neck squamous cell carcinoma (HNSCC). This study was to establish radiomics models derived from intratumoral, peritumoral, and habitat regions for identifying occult LNM in HNSCC.

**Methods:**

Patients with pathologically confirmed HNSCC from three medical Centers (from March 2014 to April 2024) and The Cancer Genome Atlas (TCGA) were enrolled. Center 1 was split into training (*n* = 330) and internal test sets (*n* = 154), while Center 2 and Center 3 served as the external test set (*n* = 183). Genomic set (*n* = 50) from TCGA and single-cell RNA sequencing set (*n* = 6) from Center 1 were used for biological analysis. We used the intratumoral, peritumoral, and habitat volumes of interest (VOIs) to extract radiomics features, respectively. Based on Logistic Regression (LR), Support Vector Machine (SVM), and Random Forest (RF) classifiers, nine radiomics models were built to confirm the optimal predictive performance. The best-performing model, along with clinical-radiologic data, was combined to develop a hybrid model. The log-rank test was used to evaluate the model’s prognostic performance. Additionally, bulk and single-cell RNA sequencing were applied for investigating the biological mechanisms underlying the optimal model.

**Results:**

The RF-habitat radiomics model showed the best performance, achieving AUCs of 0.835–0.919 across all datasets. Survival analysis further confirmed the prognostic value of the RF-habitat radiomics model. The RF-habitat radiomics model and the hybrid model notably surpassed the clinical model in predictive performance. Moreover, the RF-habitat radiomics model was associated with the abundance level of exhaustion-associated CD8 + T cells, uncovering the immune microenvironment characteristics contributing to occult LNM in HNSCC.

**Conclusions:**

The RF-habitat radiomics model demonstrated excellent performance for predicting occult LNM in HNSCC across three cohorts, providing a non-invasive solution for occult LNM. Furthermore, radiogenomic analysis further revealed the biological associations of the model, primarily related to T cell dysfunction.

**Supplementary Information:**

The online version contains supplementary material available at 10.1186/s12967-025-06474-7.

## Introduction

Head and neck squamous cell carcinoma (HNSCC) is the most common malignant neoplasm in the head and neck region, characterized by high invasiveness and metastatic potential. Despite remarkable advancements in diagnostic techniques and treatment strategies in recent years, the 5-year survival rate of patients has not significantly improved [1, 2]. This phenomenon is primarily attributed to the high heterogeneity of HNSCC, along with the high risk of local recurrence and distant metastasis. Cervical lymph node metastasis (LNM) further complicates the condition and serves as a critical determinant of poor prognosis in HNSCC [3].

Imaging examinations (ultrasound, CT, MRI, etc.), are routinely applied for assessing LN status by evaluating LN morphology and blood supply. However, a subset of patients classified as clinically LN negative (cN0) present no obvious signs on clinical and imaging evaluation. Postoperative pathological analysis often reveals that 30–40% of these cN0 patients harbor LNM [4]. The appropriate management of cN0 patients remains a topic of ongoing debate. Redaelli et al. [5] proposed routine prophylactic neck dissection for laryngeal cancer patients who were identified as T2 or higher to mitigate the risk of occult LNM. However, this approach may lead to overtreatment, with more than 70% of patients undergoing unnecessary surgery [6]. Though PET-CT is highly sensitive in assessing occult LNM, misinterpretations resulting from inflammation or infection are not uncommon [7]. Various molecular biomarkers show potential for predicting occult LNM in HNSCC, including epithelial-mesenchymal transition markers, tumor microenvironment/immune regulators, and genetic mutation signatures [8, 9]. However, their clinical translation still encounters major hurdles, particularly in standardization of detection protocols and robustness of clinical validation. Consequently, striking a balance between prophylactic neck dissection and avoiding overtreatment for cN0 patients remains a significant clinical challenge.

By transforming medical imaging data into a multitude of quantitative and complex features, radiomics provides deep insights into tumor heterogeneity and has recently gained significant attention in the medical field. Unlike conventional radiomics analysis for the whole tumor region, habitat analysis divides the tumor into subregions based on areas with similar biological features [10]. This approach offers a more accurate quantification of tumor heterogeneity. Verma et al. [11] employed habitat radiomics to MRI data of glioblastoma and identified two subregions—enhancing tumor and FLAIR hyperintensity—that had a major impact on patient prognosis, yielding a concordance index of 0.80 for predicting disease free survival. Furthermore, the two subregions were also linked to tumor aggressiveness, including tumor infiltration and hyperplastic blood vessels. Another study by Huang et al. [12] demonstrated that habitat radiomics model showed better performance than conventional radiomics model in predicting treatment response after radiofrequency ablation of colorectal cancer lung metastasis. The model identified three distinct tumor subregions—necrotic, fluid, and hypervascular—that corresponded with histopathological findings. Growing evidence highlights the close relationship between tumor subregions and biological behavior [13–15]. To date, no studies have attempted to address the clinical challenge of occult LNM through radiomics analysis of distinct tumor subregions.

Collectively, our study was intended to construct intratumoral, peritumoral, and habitat radiomics models to predict occult LNM in HNSCC. Additionally, we will further investigate the tumor microenvironment related to the radiomics model through bulk and single-cell RNA sequencing analysis.

## Materials and methods

### Patients

**Fig. 1:**
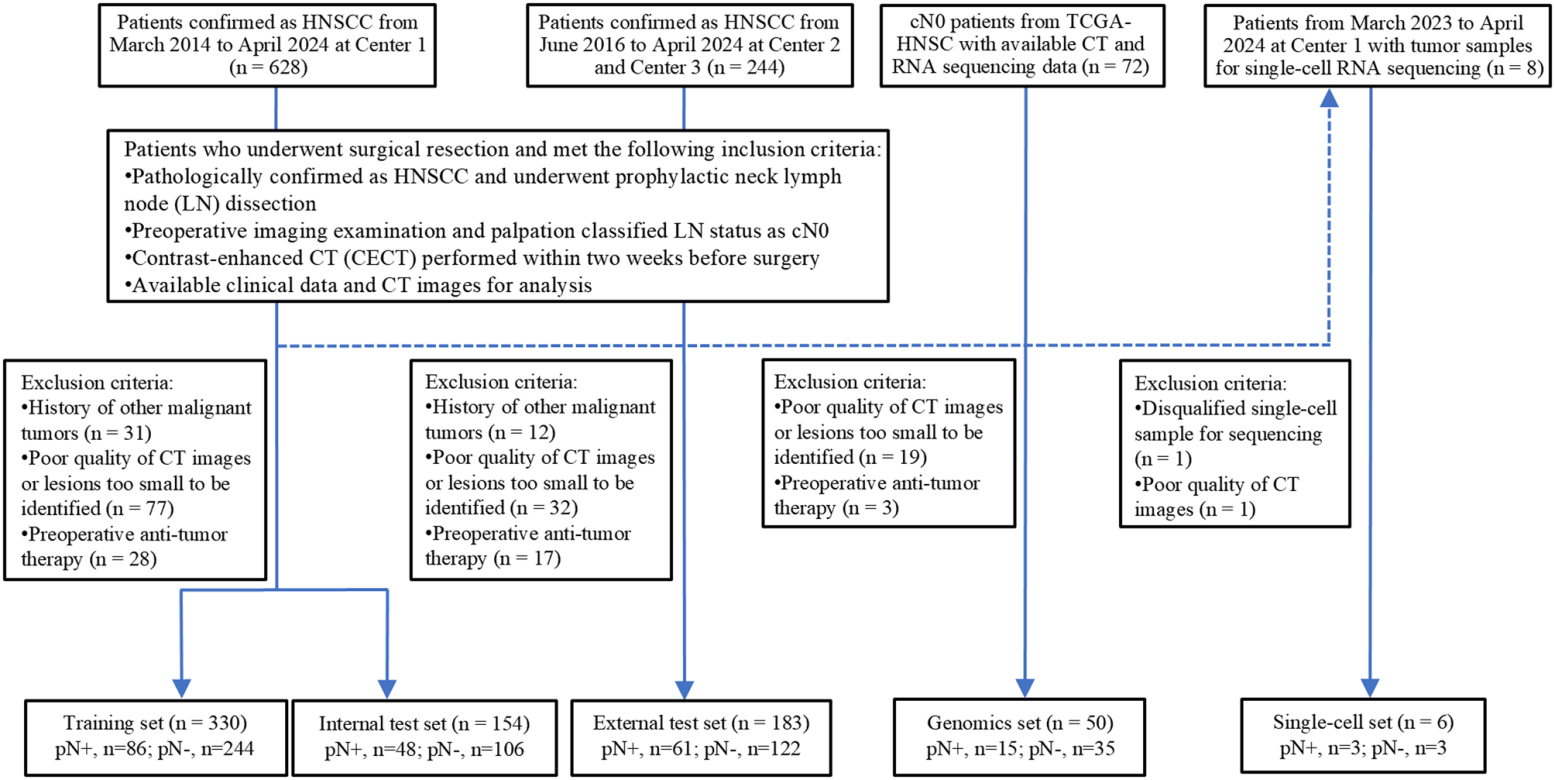
Recruitment process of HNSCC patients

This study included 723 pathologically confirmed cases of HNSCC from three Centers (from March 2014 to April 2024) and the Cancer Genome Atlas (TCGA). A total of 484 HNSCC patients from Center 1 (The First Affiliated Hospital of Chongqing Medical University) were randomly divided into a training set (*n* = 330) and an internal test set (*n* = 154) at a 7:3 ratio. Patients from Center 2 (The People’s Hospital of Hechuan) and Center 3 (Zigong Fourth People’s Hospital) served as the external test set (*n* = 183). Genomic set (*n* = 50), including available CT images from The Cancer Imaging Archive (TCIA) and RNA sequencing data from TCGA [16], were obtained to explore radiogenomic correlations. Additionally, six tumor samples from patients at Center 1 were collected for single-cell RNA sequencing. The flowchart and inclusion/exclusion criteria are detailed in Fig. 1. The study was approved by the Institutional Review Board of the First Affiliated Hospital of Chongqing Medical University (approval ID: 2021–433). Written informed consent was waived for the retrospective data sets, whereas it was acquired for single-cell sequencing set. Data collection and review adhered strictly to medical ethical standards, and this study was registered at clinicaltrials.gov (https://www.clinicaltrials.gov/; NCT06757530; January 3, 2025).

### Clinical characteristics and radiologic assessment

We comprehensively retrieved and collected patients’ clinical information, including age, sex, smoking, alcohol consumption, histological grade, and clinical T staging. Radiologic assessments were conducted by two radiologists and one otolaryngologist (P.J., J.H., F.C.M., each with 22, 6, and 8 years of experience in head and neck tumors, respectively). The assessors had no access to any clinical information and independently evaluated multi-phase CT imaging, focusing on tumor volume, maximum tumor diameter, tumor enhancement pattern, and intratumoral necrosis. The final tumor volume and maximum tumor diameter were determined by taking the mean of the measurements from three assessors, and any disagreements regarding tumor enhancement patterns or intratumoral necrosis were resolved by majority vote.

### Imaging acquisition and tumor segmentation

Image data was sourced from CT scanners at three Centers, and Supplementary Table S1 provides the specific scanning settings. After a plain scan, iodinated contrast agents were injected via the antecubital or hand vein, followed by the acquisition of venous-phase images at 65–70 s post-injection. To minimize differences in CT devices or scanning protocols, image preprocessing, including intensity normalization and resampling, was performed.

To ensure the stability of the extracted radiomic features, accurate identification and delineation of the tumor lesion are essential. With the assistance of ITK-SNAP (version 3.8.0), a widely recognized software for medical image segmentation, reader 1 (J.H.) meticulously depicted the region of interest (ROI) of the tumor on each CT slice. Subsequently, we overlaid the ROIs from all slices to form a three-dimensional (3D) volume of interest (VOI), providing a precise spatial foundation for subsequent analysis. After four weeks, reader 1 (J.H.) and reader 2 (C.X.W., with 6 years of experience in head and neck tumors) independently delineated tumor lesion in a random sample of 30 cases to assess inter- and intraobserver variability. Any uncertainties in tumor segmentation were resolved through consensus review with a senior doctor (P.J.).

## Sub-region generation

### Division of peri- and intratumoral areas

**Fig. 2:**
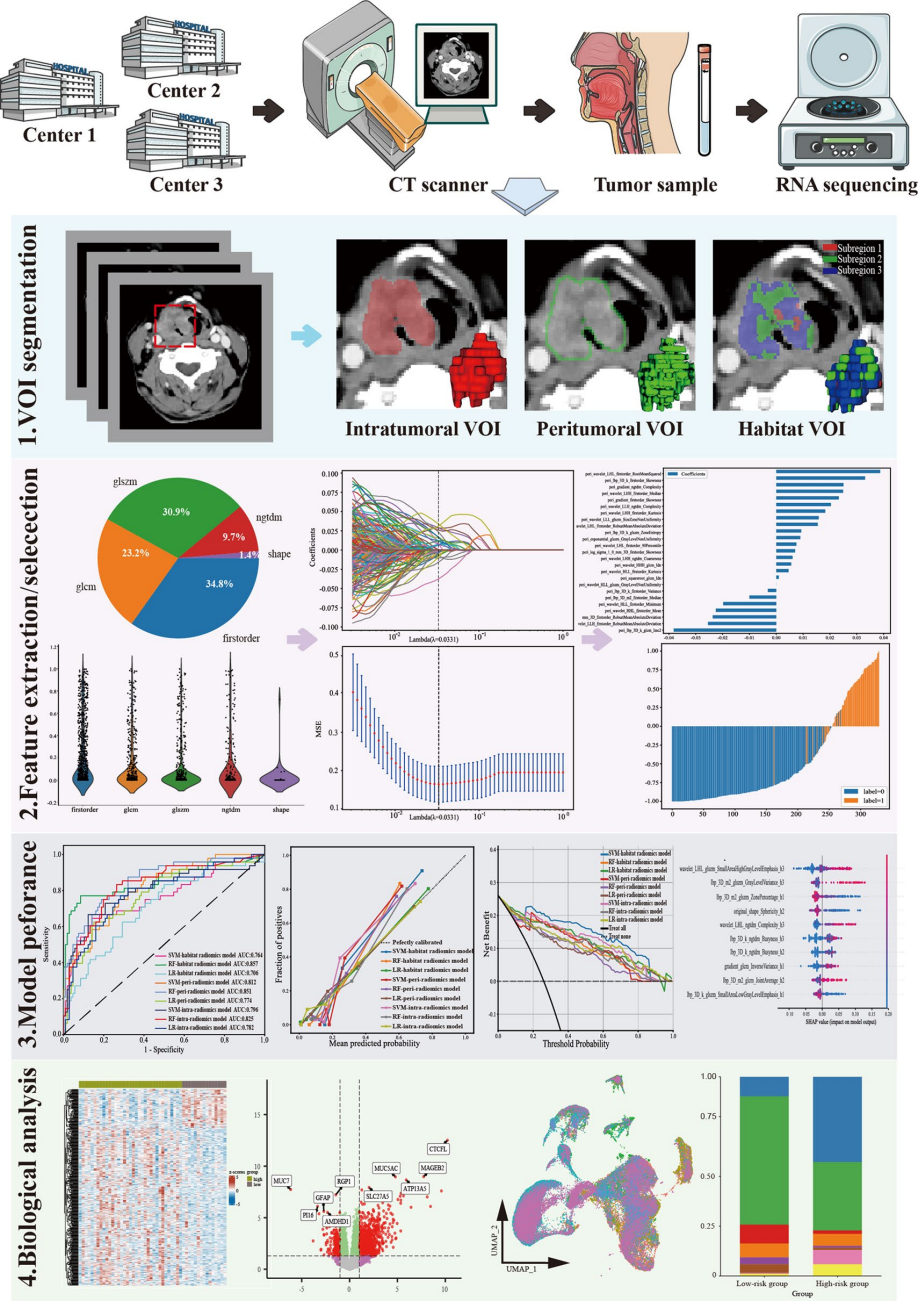
The framework of this retrospective study. Step 1: VOI segmentation. The intratumoral, peritumoral, and habitat VOIs were derived from tumor delineation on the venous-phase CT images. Step 2: Feature extraction/section. Step 3: Model building and evaluation. LR, SVM, and RF classifiers were employed to construct predictive models. Step 4: Biological analysis. Bulk and single-cell RNA sequencing analysis were performed to explore the biological relationship

After obtaining the tumor’s VOI, we isotropically expanded the delineated ROIs of each VOI outward by 1 mm to define the peritumoral region. The choice of expansion length for the peritumoral region was determined based on preliminary experiments using different distances, with 1 mm peritumoral distance ultimately demonstrating the best result (Supplementary Figure S1), which is consistent with other literature [17–19]. The expanded peritumoral regions were stacked to form the peritumoral VOI. Any abnormal regions, such as cavities or extracapsular areas, were manually adjusted. The original VOIs were considered as the intratumoral region, which exclusively encompassed the core tumor area without including surrounding tissues. Both the intratumoral and peritumoral VOIs were used for subsequent radiomics analyses. The study flowchart is presented in Fig. 2.

### Habitat region clustering

To further quantify the heterogeneity of different tumor regions, we calculated 18 radiomics features for each voxel within the tumor VOIs, which include quantitative descriptions of image grayscale distribution, spatial relationships, and texture patterns. Detailed explanations of the 18 features are provided in Supplementary Table S2. The K-means algorithm, a commonly used unsupervised clustering algorithm, was exploited to automatically sort the processed voxels into individual subregions. This method performs unsupervised classification of the radiomics feature information for each voxel, dividing the VOIs into distinct subregions based on feature similarity. In addition, we tested cluster numbers from 2 to 10 and utilized the Davis-Bouldin (DB) score [20] to identify the most suitable partitioning of tumor subregions.

### Radiomics feature extraction and model development

Based on the intratumoral, peritumoral, and habitat VOIs, radiomics features were separately extracted through the Python package PyRadiomics. To ensure consistency and reliability, features with intra- and interclass correlation coefficients (ICCs) > 0.75 were retained and subjected to Z-score normalization. The Mann-Whitney *U* test was conducted to determine statistically discrepant features, while Spearman correlation analysis was performed to remove highly correlated features (*r* > 0.9). The remaining features were chosen using the Least Absolute Shrinkage and Selection Operator (LASSO) method. Finally, founded on the optimal features of intratumoral, peritumoral, and habitat VOIs, models were constructed by using Logistic Regression (LR), Support Vector Machine (SVM), and Random Forest (RF) classifiers to differentiate between cN0 patients with and without LNM. The stability and generalizability of the model were assessed using 5-fold cross-validation.

In the training set, uni- and multivariable LR analyses were conducted between pathological LN positive (pN+) and pathological LN negative (pN-) to identify independent predictive factors among clinical characteristics, radiological indicators, and radiomics scores (the output of the best model). The Variables with *P* < 0.05 were incorporated into the construction of a hybrid model. Additionally, regarding the results from uni- and multivariate analyses, a clinical model that integrated significant clinical characteristics and radiological indicators were built.

### Prognostic evaluation and biological functions exploration

The cutoff value (0.270) was calculated by the maximum Youden index in the area under the receiver operating characteristic (ROC) curve (AUC) analysis of the training set. Patients were categorized into high- and low-risk groups according to the cutoff value. The log-rank test was used to compare 3-year disease-free survival (DFS) and overall survival (OS) between the two groups. Further details on the follow-up strategy can be found in Appendix S1. Additionally, differentially expressed genes (DEGs) between the low- and high-risk groups in the genomic set were analyzed by means of the R package DESeq2. Subsequently, Kyoto Encyclopedia of Genes and Genomes (KEGG) analysis, Gene Set Enrichment Analysis (GSEA), and Gene Set Variation Analysis (GSVA) were performed using the R package clusterProfiler. Furthermore, single-cell RNA sequencing was utilized to explore cell composition and functional state of the tumor heterogeneity between the two groups. To validate these cellular and molecular findings, tumor samples from both metastasis and non-metastasis groups were employed for multiplex immunofluorescence (mIF) staining to assess the distribution of key cell populations and the expression of important molecules at the tissue level. Supplementary Appendix S1 provides details on the radiotranscriptomic analysis.

### Statistical analysis

Statistical analyses were conducted using SPSS (version 24.0) and Python (version 3.8) software. Continuous variables were expressed as mean ± standard deviation and compared between pN + group and pN- group using *t*-tests or Mann-Whitney *U* tests. Categorical variables were presented as frequencies and percentages, and compared between the two groups using chi-square tests or Fisher’s exact tests. *P* value < 0.05 was considered statistically significant. Additionally, the AUC was calculated to assess the diagnostic performance, and DeLong method was employed to compare models’ performance. Calibration curves and decision curves analysis were used to evaluate models’ performance and clinical applicability.

## Results

### Patient characteristics

The incidence of occult LNM was 26.06% (86 of 330) in the training set, 31.17% (48 of 154) in the internal test set, 33.33% (61 of 183) in the external test set, and 30.00% (15 of 50) in the genomic set, and the LN status was balanced across the four cohorts (*P* = 0.337). The clinical characteristics of the study population is summarized in Table 1. Grouping by LN status in the training set revealed that pN + patients were more likely to exhibit T3 or T4 stages, poor differentiation, and imaging features such as larger tumor volume, longer maximum tumor diameter, and a greater tendency for heterogeneous enhancement and tumor necrosis (all *P* < 0.05). On the other hand, there were no significant differences between the two groups in terms of age, sex, smoking, and alcohol consumption (Table 2).

**Table 1:** Baseline clinical characteristics of the patients in the datasets

Clinical characteristics	Training set (*n* = 330)			Internal test set (*n* = 154)			External test set (*n* = 183)				TCGA set (*n* = 50)		
	pN- (*n* = 244)	pN+ (*n* = 86)	*P*	pN- (*n* = 106)	pN+ (*n* = 48)	*P*	pN- (*n* = 122)	pN+ (*n* = 61)	*P*		pN- (*n* = 35)	pN+ (*n* = 15)	*P*
Age (mean ± SD, years)	62.48 ± 8.73	61.50 ± 7.95	0.344	61.15 ± 8.83	63.21 ± 9.26	0.189	62.34 ± 8.78	62.43 ± 10.17	0.955		61.23 ± 11.60	61.00 ± 8.02	0.945
Sex			0.473			0.006*			0.005*				0.882
Male	236(96.7)	81(94.2)		103(97.2)	40(83.3)		112(91.8)	46(75.4)			28(80.0)	11(73.3)	
Female	8(3.3)	5(5.8)		3(2.8)	8(16.7)		10(8.2)	15(24.6)			7(20.0)	4(26.7)	
Smoking			0.736			0.010*			0.001*				0.933
Yes	226(92.6)	78(90.7)		100(94.3)	38(79.2)		106(86.9)	40(65.6)			30(85.7)	12(80.0)	
No	18(7.4)	8(9.3)		6(5.7)	10(20.8)		16(13.1)	21(34.4)			5(14.3)	3(20.0)	
Alcohol consumption			0.934			0.281			0.149				0.794
Yes	170(69.7)	61(70.9)		77(72.6)	30(62.5)		79(64.8)	32(52.5)			24(68.6)	9(60.0)	
No	74(30.3)	25(29.1)		29(27.4)	18(37.5)		43(35.2)	29(47.5)			11(31.4)	6(40.0)	
Histological grade			0.002*			0.259			0.754				0.089
Poor	34(13.9)	26(30.2)		9(8.5)	7(14.6)		12(9.8)	5(8.2)			9(25.7)	0(0.0%)	
Moderate	117(48.0)	38(44.2)		46(43.4)	24(50.0)		57(46.7)	26(42.6)			17(48.6)	9(60.0)	
Well	93(38.1)	22(25.6)		51(48.1)	17(35.4)		53(43.5)	30(49.2)			9(25.7)	6(40.0)	
Clinic T stage			< 0.001*			< 0.001*			0.021*				0.982
T1	75(30.7)	13(15.1)		24(22.6)	2(4.2)		37(30.3)	19(31.1)			3(8.5)	1(6.7)	
T2	88(36.1)	23(26.7)		30(28.3)	5(10.4)		31(25.4)	9(14.8)			10(28.6)	4(26.7)	
T3	63(25.8)	31(36.1)		46(43.4)	26(54.2)		49(40.2)	23(37.7)			10(28.6)	4(26.6)	
T4	18(7.4)	19(22.1)		6(5.7)	15(31.2)		5(4.1)	10(16.4)			12(34.3)	6(40.0)	
Radiologic features-tumor volume (cm^3^)	6.36 ± 5.01	9.95 ± 6.99	< 0.001*	4.48 ± 1.52	15.98 ± 17.16	< 0.001*	5.11 ± 1.59	12.66 ± 9.42	< 0.001*		11.17 ± 9.37	7.65 ± 8.86	0.165
Radiologic features-maximum tumor diameter (cm)	2.76 ± 0.95	3.72 ± 1.05	< 0.001*	2.30 ± 0.63	4.06 ± 1.49	< 0.001*	2.52 ± 0.66	3.86 ± 1.19	< 0.001*		3.16 ± 1.42	2.75 ± 2.05	0.418
Radiologic features-enhancement patterns			0.019*			< 0.001*			0.020*				0.933
Homogenous enhancement	187(76.6)	54(62.8)		88(83.0)	23(47.9)		92(75.4)	35(57.4)			30(85.7)	12(80.0)	
Heterogeneous enhancement	57(23.4)	32(37.2)		18(17.0)	25(52.1)		30(24.6)	26(42.6)			5(14.3)	3(20.0)	
Radiologic features-intratumoral necrosis			0.022*			0.934			0.507				0.933
Yes	32(13.1)	21(24.4)		9(8.5)	5(10.4)		16(13.1)	11(18.0)			5(14.3)	3(20.0)	
No	212(86.9)	65(75.6)		97(91.5)	43(89.6)		106(86.9)	50(82.0)			30(85.7)	12(80.0)	

Abbreviations: TCGA, the Cancer Genome Atlas; SD, standard deviation; pN-, Pathological LN negative; pN+, Pathological LN positive

*P < 0.05

### Habitat region and feature selection

After applying the K-means method with different numbers of clusters, the optimal DB score was observed with 3 clusters (Supplementary Figure S2). Therefore, the habitat VOIs with 3 subregions were used for subsequent habitat analysis. There were 1034 extracted radiomics features corresponding to the intratumoral, peritumoral, and habitat VOIs, respectively. Of these, 880 features were those with ICCs > 0.75 and were retained to maintain feature reproducibility. Notably, ICCs are unsuitable for evaluating unsupervised habitat radiomics features. Subsequently, the features were further filtered using the Mann-Whitney *U* test, Spearman correlation, and LASSO method. Ultimately, 28 features from the intratumoral VOIs (Supplementary Figure S3), 25 features from the peritumoral VOIs (Supplementary Figure S4), and 31 features from the habitat VOIs (Supplementary Figure S5) were retained for modeling, respectively.

### Performance evaluation of all predictive models

**Fig. 3:**
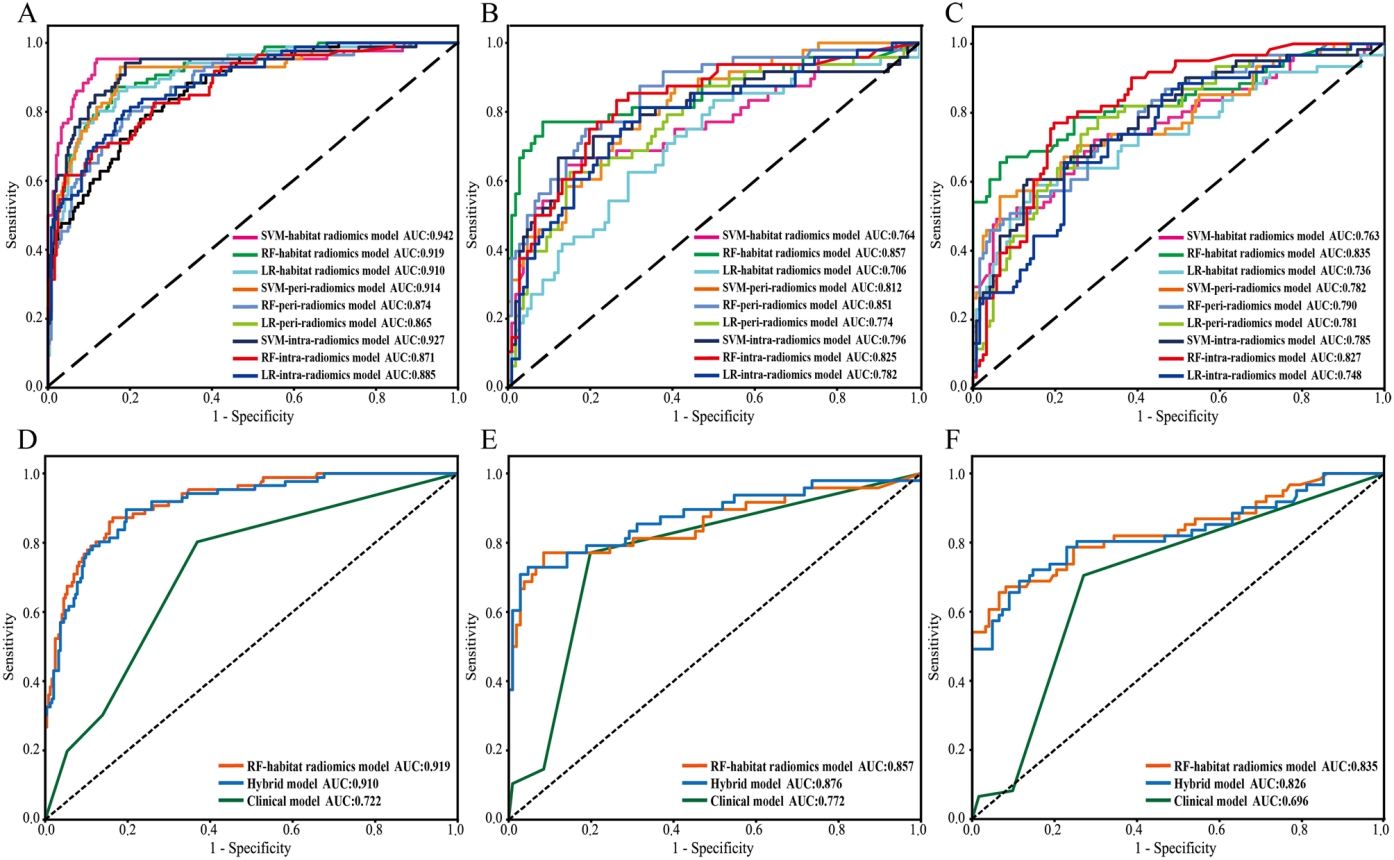
Performance evaluation of different models for predicting occult LNM in HNSCC patients. ROC curves for intratumoral, peritumoral, and habitat radiomics models in the training (**A**), internal test (**B**), and external test sets (**C**), respectively. Additionally, the predictive performances of the RF-habitat radiomics model, clinical model, and hybrid model in the in the training (**D**), internal test (**E**), and external test sets (**F**), respectively

**Fig. 4:**
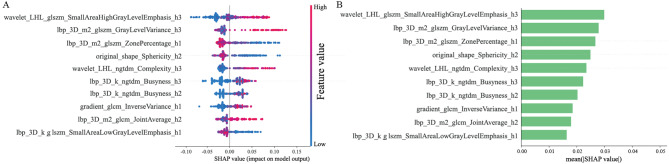
Model interpretability of the RF-habitat radiomics model through SHAP plot. (**A**) The SHAP bar chart showed the importance of top 10 features in the model. (**B**) The SHAP bees-warm plot showed the positive or negative effects of each feature through red and blue colours

Using LR, SVM, and RF classifiers, nine radiomics models were constructed based on the optimal features extracted from intratumoral, peritumoral, and habitat VOIs. Among them, the RF-based radiomics model demonstrated superior performance than the LR- and SVM-based models in two test sets, with the RF-habitat radiomics model exhibiting excellent results (Supplementary Table S3 and Fig. 3A–C). It achieved AUCs of 0.919 (95% confidence interval [CI]: 0.886–0.951) in the training set, 0.857 (95% CI: 0.780–0.933) in the internal test set, and 0.835 (95% CI: 0.765–0.904) in the external test set. Using Shapley additive explanations (SHAP) method for visualization, the RF-habitat radiomics model’s contribution of individual features was demonstrated (Fig. 4). Among the top 10 features, four originated from region 3, three from region 2, and three from region 1, with features of region 3 showing higher weight in the model development. Moreover, a significant difference in RF-habitat radiomics scores was detected between the pN + and pN- groups in the training set (Table 2). The ROC curves for all predictive models are shown in Fig. 3, and the calibration curves and decision curves analysis are provided in Supplementary Figures S6 and S7.

Independent predictors of occult LNM, as determined by uni- and multivariate LR analyses, were maximum tumor diameter (odds ratio [OR] = 0.523), histological grade (OR = 0.345), and RF-habitat radiomics score (OR = 6.297) (Table 2). Using these three variables, we constructed a hybrid model to estimate LN status, with AUCs of 0.910 (95% CI: 0.874–0.944), 0.876 (95% CI: 0.806–0.944), 0.826 (95% CI: 0.753–0.898) in the training, internal test and external test sets, respectively (Table 3). Additionally, we found that the hybrid model and the RF-habitat radiomics model significantly outperformed the clinical model, which incorporated maximum tumor diameter and histological grade (both *P* < 0.05) (Supplementary Figure S8).

**Table 2:** Uni- and multivariate LR analyses of variables for predicting occult LNM in patients in the training set

Clinical characteristics	Univariate analysis			Multivariate analysis		
	OR	95% CI	*P*	OR	95% CI	*P*
Age	0.987	0.959–1.015	0.360			
Sex	0.549	0.175–1.727	0.305			
Smoking	0.777	0.325–1.857	0.570			
Alcohol consumption	1.062	0.619–1.822	0.827			
Histological grade						
Poor	Ref			Ref		
Moderate	0.515	0.270–0.984	0.045*	0.345	0.123–0.963	0.042*
Well	0.415	0.207–0.832	0.013*	0.622	0.217–1.787	0.378
Clinical T stage						
T1	Ref			Ref		
T2	1.508	0.715–3.181	0.281	0.979	0.343–2.799	0.969
T3	2.839	1.369–5.886	0.005*	0.774	0.238–2.516	0.670
T4	6.090	2.543–14.581	< 0.001*	1.123	0.224–5.644	0.888
Radiologic features-tumor volume	1.121	1.066–1.178	< 0.001*	0.911	0.829–1.001	0.052
Radiologic features-maximum tumor diameter	2.414	1.851–3.148	< 0.001*	0.523	0.278–0.982	0.044*
Radiologic features-enhancement pattern	1.944	1.146–3.297	0.014*	0.783	0.289–2.119	0.630
Radiologic features-intratumoral necrosis	2.140	1.155–3.965	0.016*	2.243	0.855–5.882	0.101
RF-Habitat radiomics score	3.338	2.572–4.330	< 0.001*	6.297	3.988–9.943	< 0.001*

Abbreviations: RF, Random Forest; HR: hazard ratio; CI: confidence interval

*P < 0.05

**Table 3:** The performance of predictive models in predicting occult LNM

Cohort	Model	AUC (95%CI)	Accuracy	Sensitivity	Specificity	PPV	NPV
Training set	RF-habitat radiomics model	0.919 [0.886–0.951]	0.842	0.860	0.836	0.649	0.944
	Clinical model	0.722 [0.665–0.779]	0.715	0.302	0.861	0.433	0.778
	Hybrid Model	0.910 [0.874–0.944]	0.824	0.884	0.803	0.613	0.951
Internal test set	RF-habitat radiomics model	0.857 [0.780–0.933]	0.864	0.750	0.915	0.800	0.890
	Clinical model	0.772 [0.696–0.846]	0.675	0.146	0.915	0.437	0.703
	Hybrid Model	0.876 [0.806–0.944]	0.877	0.708	0.953	0.872	0.878
External test set	RF-habitat radiomics model	0.835 [0.765–0.904]	0.836	0.639	0.934	0.830	0.838
	Clinical model	0.696 [0.622–0.769]	0.628	0.082	0.902	0.294	0.663
	Hybrid Model	0.826 [0.753–0.898]	0.814	0.672	0.885	0.745	0.844

Abbreviations: RF, Random Forest; AUC, area under the curve; CI, confidence interval; PPV, positive predictive value; NPV, negative predictive value

### Clinical outcomes and biological functions related to the RF-habitat radiomics model

**Fig. 5:**
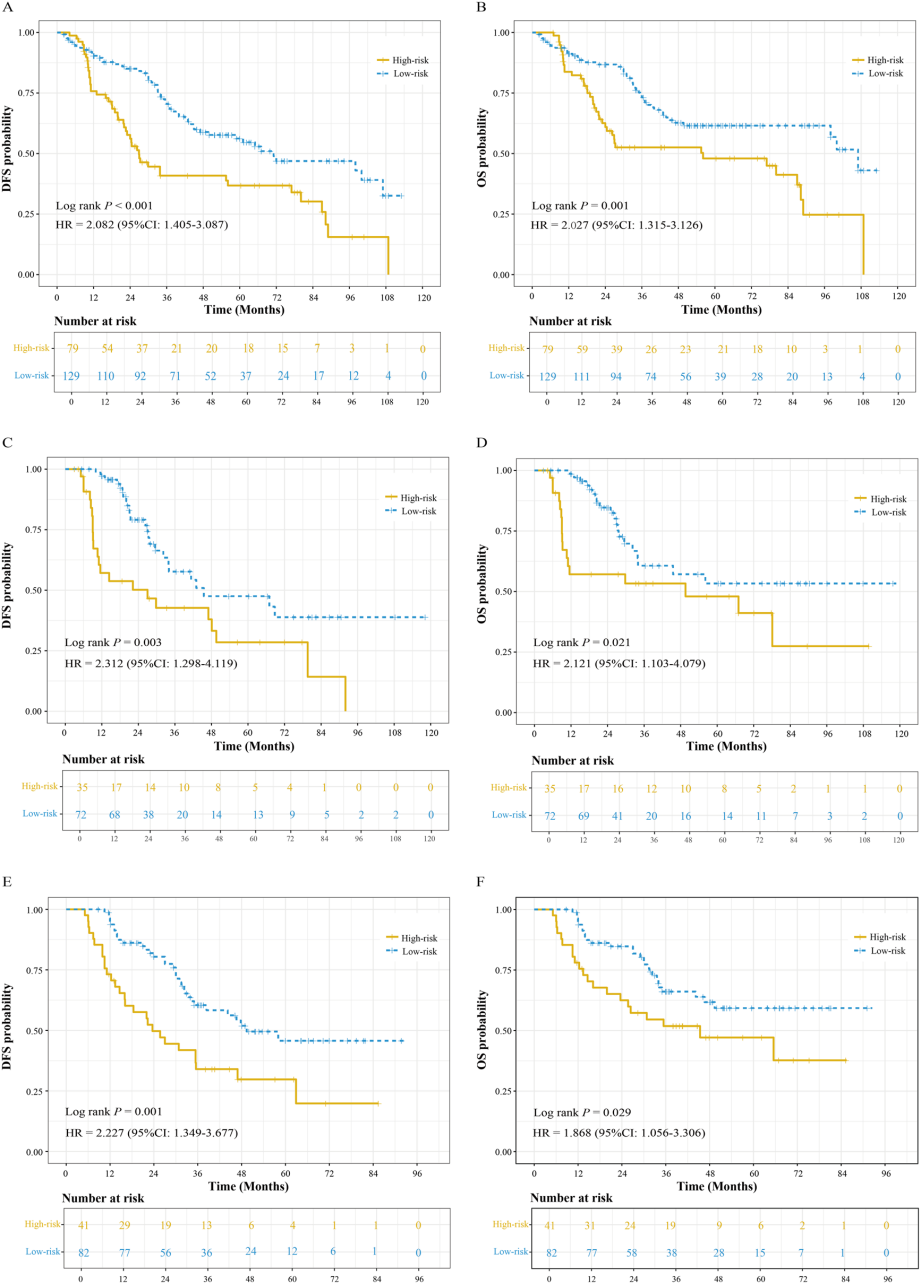
Kaplan-Meier analyses of 3-year DFS and OS stratified by the RF-habitat radiomics model. (**A**, **B**) Training set. (**C**, **D**) Internal test set. (**E**, **F**) External test set

We collected patients’ 3-year DFS and OS data to evaluate the prognostic value of the RF-habitat radiomics model. In the training cohort (208 eligible patients), the 3-year DFS and OS rates were 58.1% and 65.9% in the low-risk group, while the 3-year DFS and OS rates in the high-risk group were 39.2% and 49.4%, respectively. In the internal test set (107 eligible patients), the 3-year DFS and OS rates were 65.3% and 72.2% in the low-risk group, compared to 37.1% and 51.4% in the high-risk group. Similarly, in the external test set (123 eligible patients), the 3-year DFS and OS rates were 58.5% and 67.1% in the low-risk group, compared to 31.7% and 48.8% in the high-risk group (Supplementary Table S4). Survival analysis further confirmed that patients stratified by the RF-habitat radiomics model had significantly different prognoses across all sets (Fig. 5).

**Fig. 6:**
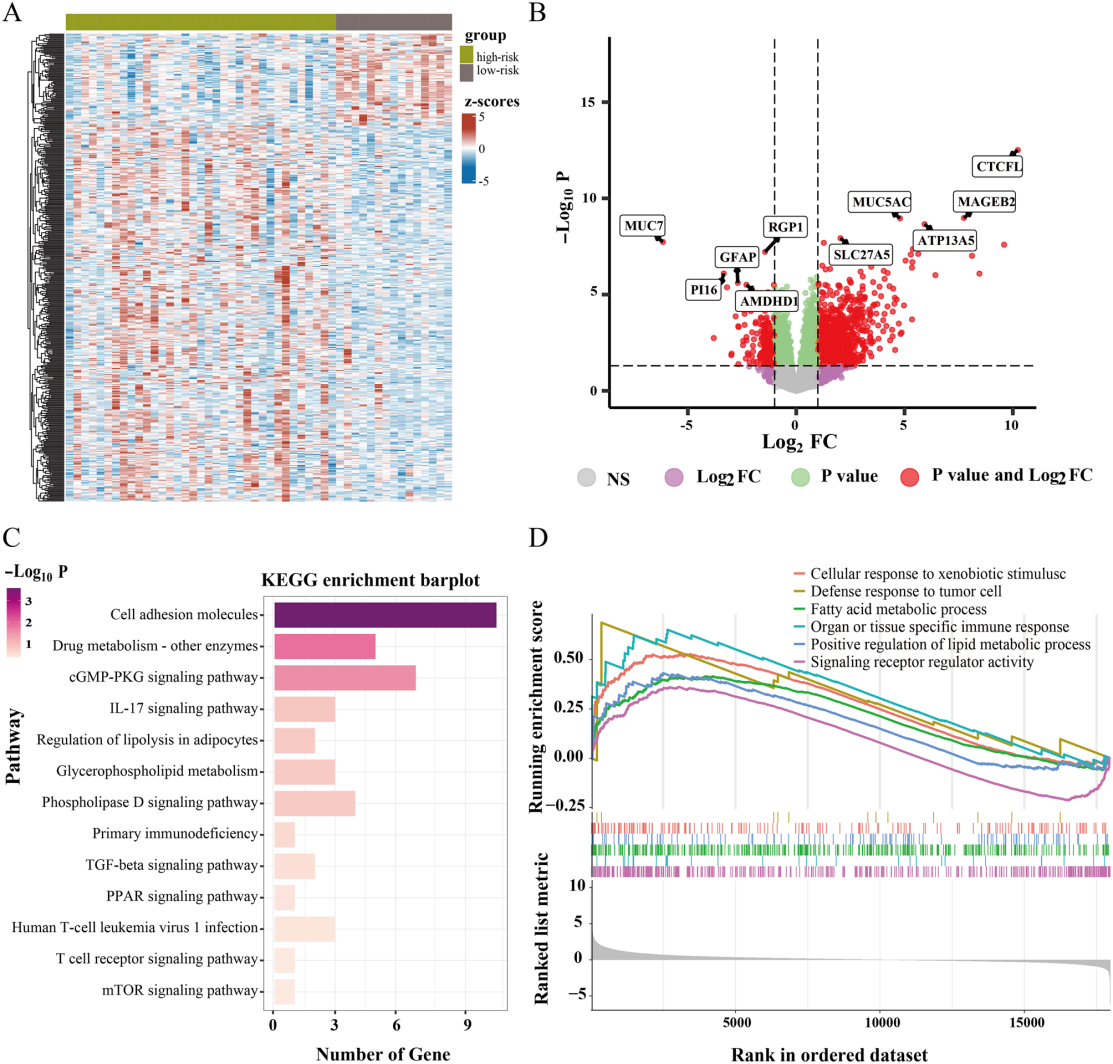
Bulk RNA sequencing analysis for biological basis associated with the RF-habitat radiomics model. (**A**) Heatmap depicted the expression profiles of DEGs between low and high-risk groups stratified by the RF-habitat radiomics model. (**B**) Volcano plot showed the distribution of DEGs between two groups. (**C**) KEGG enrichment barplot displayed the key pathways enriched by DEGs. (**D**) GSEA based on the Gene Ontology (GO) database revealed immune and metabolism-related pathways enriched in the high-risk group

Four hundred sixty-three DEGs were identified in the genomic set between low- or high-risk groups (Fig. 6A, B). KEGG results showed that DEGs were closely associated with immune-regulatory pathways, including T cell receptor signaling pathway, primary immunodeficiency, and IL-17 signaling pathway. Pathways related to metabolism and signal transduction were also upregulated in high-risk groups (Fig. 6C). Similarly, GSEA indicated that immune regulation pathways and lipid metabolism-related pathways were significantly enriched in the high-risk group (Fig. 6D).

**Fig. 7:**
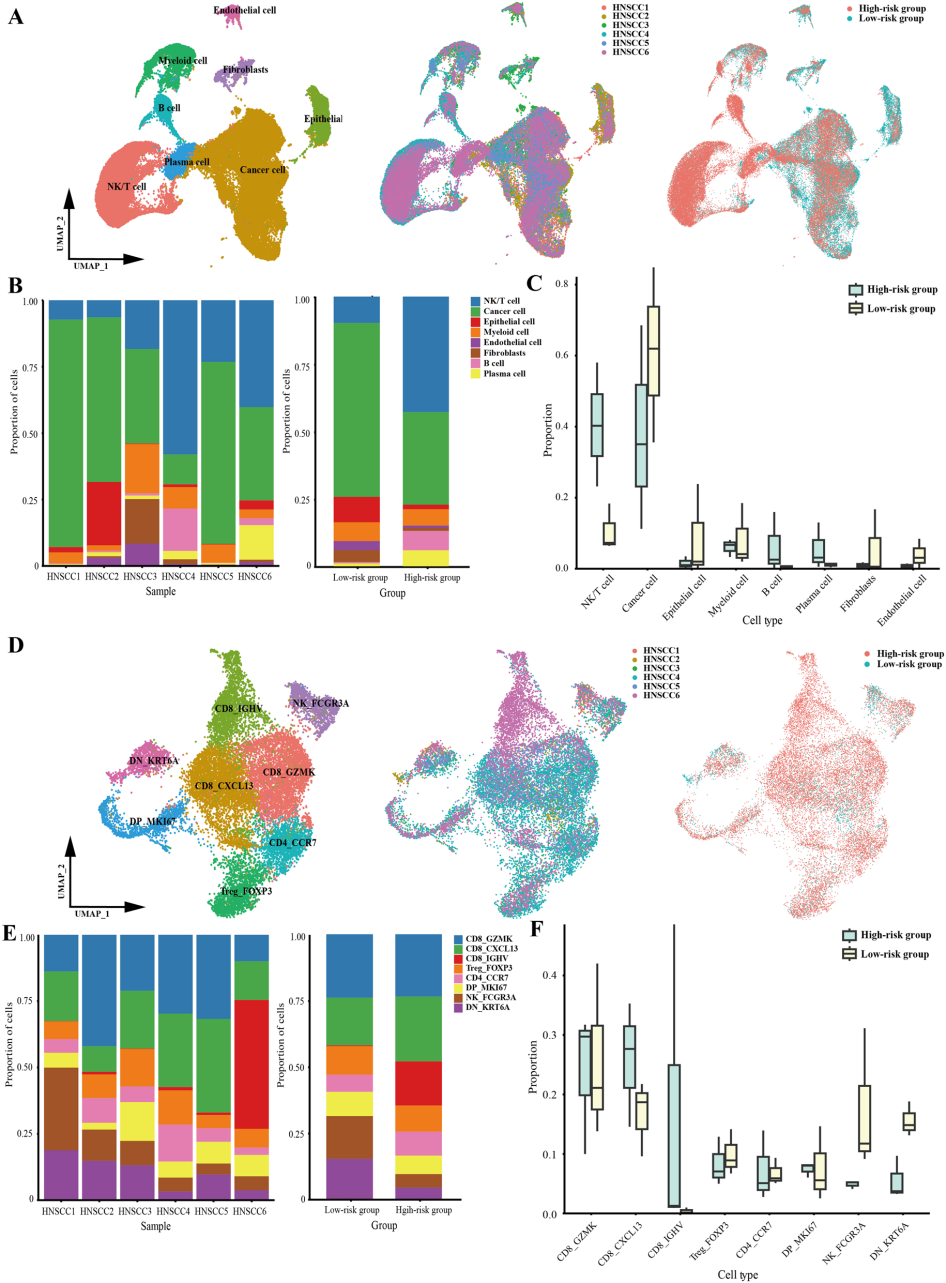

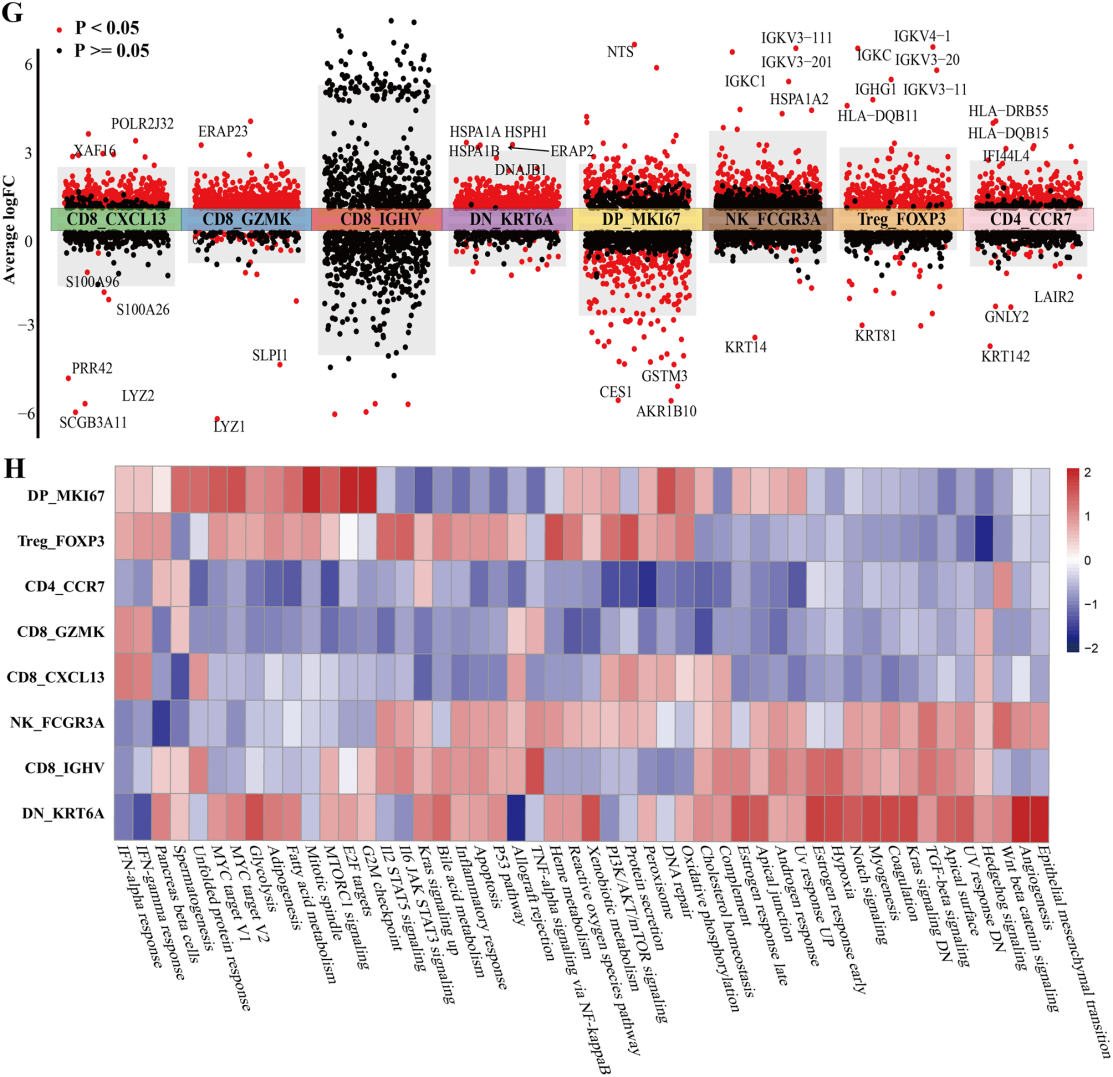
Single-cell landscape of tumor microenvironment related to the RF-habitat radiomics model. (**A**) Uniform manifold approximation and projection (UMAP) plots color coded for cell types (left), individual samples (middle), and groups (right) (low-risk group: HNSCC1-HNSCC3; high-risk group: HNSCC4 − HNSCC6). (**B**) Proportions of eight identified cell types presented in bar plots across different samples (left) and groups (right). (**C**) The infiltration levels of eight cell types in low- and high-risk groups. (**D**) UMAP plots displayed NK/T cells regrouped into eight subgroups. (**E**) Proportions of each subgroup presented in bar plots across different samples (left) and groups (right). (**F**) The infiltration levels of each subgroup in low- and high-risk groups. (**G**) DGEs analysis showed up- and down-regulated genes of each subgroup. (**H**) GSVA of Hallmark gene sets exhibited the pathway activity profiles across eight subgroups. Up- and downregulated signaling pathways were shown in red and blue, respectively

**Fig. 8:**
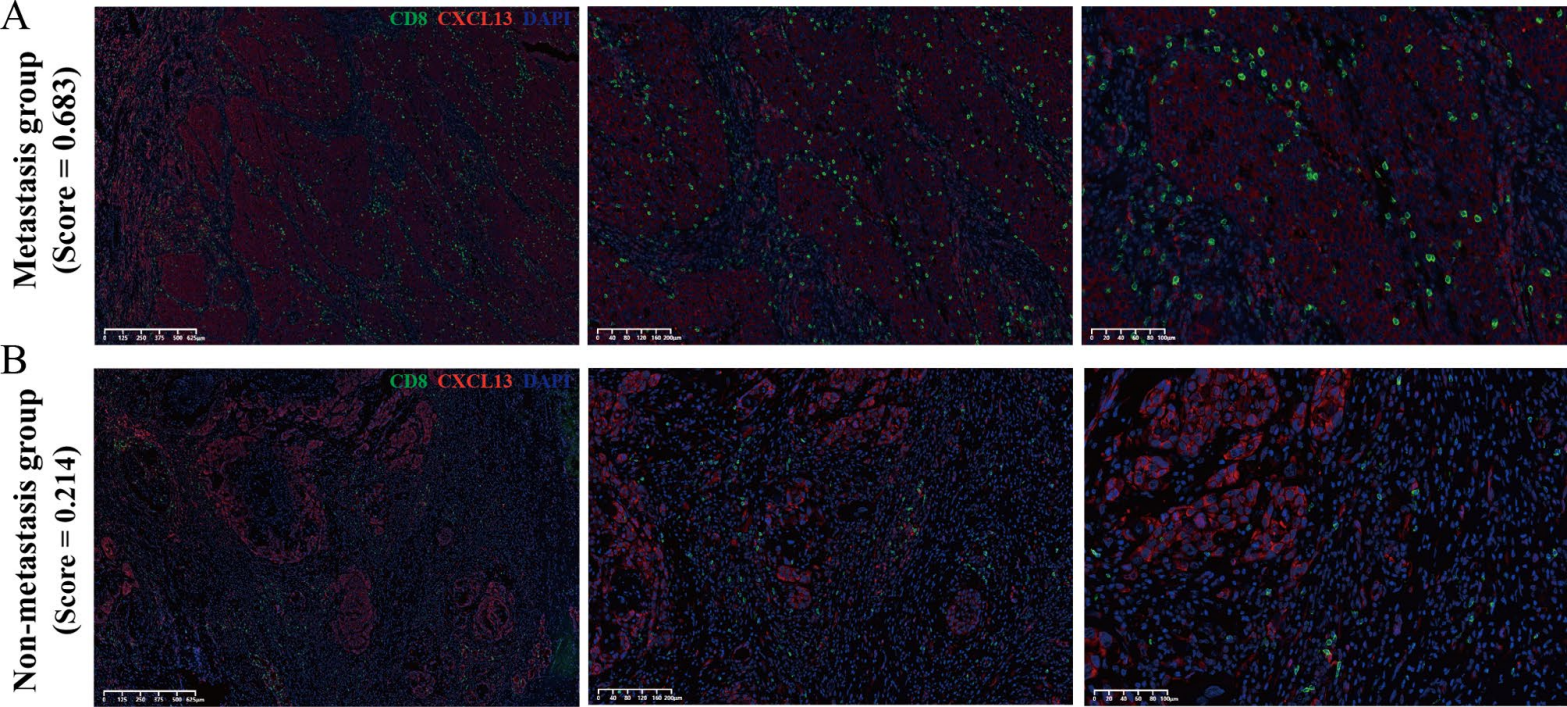
MIF results for CD8_CXCL13 cells in pN + and pN- groups. (**A**) One cN0 patient with HNSCC confirmed as pN+ (score = 0.683) was infiltrated by abundant CD8_CXCL13 cells in tumor microenvironment. (**B**) Another cN0 patient with HNSCC confirmed as pN- (score = 0.214) showed minimal CD8 + T cell infiltration. Green fluorescent label CD8; Red fluorescent label CXCL13; DAPI blue fluorescent label cell nuclei

We conducted single-cell RNA sequencing analysis on 6 HNSCC patients grouped by the RF-habitat radiomics model. After strict quality control, we obtained 68,613 cells in total, which were identified as epithelial cells, endothelial cells, fibroblasts, myeloid cells, NK/T cells, B cells, and plasma cells (Fig. 7A–C). The top five marker genes of each cluster are displayed in Supplementary Figure S9A. Further, malignant epithelial cells were classified as cancer cells using chromosomal copy number variation profile analysis (inferCNV) with B cells as reference (Supplementary Figure S10). Cell populations were unevenly distributed both across samples and between groups, particularly with a distinct difference in the infiltration rate of NK/T cells. It indicated that the high-risk group appeared a more complex immune microenvironment, partially aligned with the findings of bulk RNA sequencing analysis (Fig. 7D–F). Subsequently, we regrouped NK/T cells as CD8_GZMK, CD8_CXCL13, CD8_IGHV, CD4_CCR7, Treg_FOXP3, NK_FCGR3A, DN_KRT6A, DP_MKI67. The top five marker genes across T/NK cell subclusters are displayed in Supplementary Figure S9B. Notably, it showed that the infiltration levels of CD8_CXCL13 and CD8_IGHV cells were heightened in the high-risk group. Though not statistically significant, the two subtypes appeared to be key components contributing to the differences and complexity of the immune microenvironment between the two groups. Further analysis indicated that the high density of CD8_IGHV cells was primarily driven by the imbalanced proportion of the HNSCC6 sample. In contrast, exhaustion-associated CD8_CXCL13 cells exhibited a relatively stable higher abundance, which is consistent with previous research findings [21–23]. Additionally, the CD8_CXCL13 cells proportion derived from the CIBERSORTx deconvolution algorithm of 50 bulk RNA data also showed results consistent with single-cell analysis (Supplementary Figure S11). Collectively, it indicated that exhaustion-associated CD8_CXCL13 cells may be pivotal in the immune microenvironment contributing to occult LNM in HNSCC. To further elucidate the molecular characteristics and functional states, we performed DGEs analysis for each subgroup of NK/T cells, as shown in Fig. 7G. Moreover, GSVA revealed that CD8_CXCL13 cells were mainly associated with upregulation of the INF-alpha or gamma response signaling pathway (Fig. 7H). MIF analysis demonstrated that the metastasis group exhibited a relatively higher abundance of exhaustion-associated CD8 + T cells (CD8_CXCL13) (Fig. 8).

## Discussion

In this study, the RF-habitat radiomics model outperformed both intratumor and peritumor radiomics models in predicting occult LNM in HNSCC, showing outstanding predictive capability. By quantifying distinct tumor subregions, the model offered a more nuanced understanding of tumor heterogeneity. Furthermore, the RF-habitat radiomics score effectively stratified HNSCC patients in terms of 3-year DFS and OS. Both the RF-habitat radiomics model and the hybrid model surpassed the clinical model in predictive performance. Remarkably, radiogenomic analysis suggested that the RF-habitat radiomics model was linked to exhaustion-associated CD8 + T cell, revealing the immune microenvironment characteristics potentially contributing to occult LNM.

Recently, the rapid development of radiomics has brought a novel and promising perspective for LNM prediction in head and neck tumors. Zhang and his team [24] proposed that a clinical-radiomics nomogram based on iodine maps achieved high predictive accuracy in identifying LNM for HNSCC patients. In addition, a study by Wang and his team [15] collected data from 553 cN0 patients with laryngeal squamous cell carcinoma (LSCC) and compared the performance of deep learning, radiomics, and hybrid models in identifying LN status. It showed that the decision-based hybrid model realized superior predictive accuracy across all study cohorts. Nevertheless, most existing literature primarily concentrates on the features of whole lesion, which may not fully capture the microenvironmental characteristics of tumor’s subregions associated with LNM.

Habitat radiomics decomposes tumors into multiple “habitat” regions with distinct biological characteristics and has gradually become an innovative approach for in-depth analysis of tumor heterogeneity. Wei et al. [25] used multi-sequence MRI data to manually segment the tumor and edema regions for predicting MGMT promoter methylation in grade II-IV astrocytoma. Their results showed that a hybrid model combining two subregions surpassed the one-region models, achieving the best results. However, the manual subregion segmentation process is tedious and heavily relied on radiologists’ subjective experience [26]. Surprisingly, this study applied the K-means method, a classic non-supervised clustering technique, to segregate tumor regions according to voxel feature similarity, which reduced manual intervention and improved the reproducibility of the results [27, 28]. Moreover, unlike previous studies that used imaging grayscale values for clustering, the study calculated 18 radiomics features for tumor voxels, providing a more comprehensive and detailed description of the tumor’s heterogeneity [12, 29]. Our findings revealed that the RF-habitat radiomics model showed excellent precision in the prediction of occult LNM, offering valuable support for preoperative stratification of HNSCC patients. The habitat subregions identified in this study include three areas: region 3, tumor enhancement zone; region 2, tumor necrotic zone; and region 1, transitional zone between region 2 and 3. Among them, region 3 played the most crucial role in model construction and classification. This subregion, corresponding to the area with the most pronounced tumor enhancement on macroscopic appearance, likely reflects increased blood perfusion due to neovascularization, which has been proven to be strongly connected to early metastasis and high invasiveness [30–33]. Therefore, habitat radiomics achieved a more detailed and precise description of tumor heterogeneity for detecting occult LNM in HNSCC by clustering tumor into subregions.

Both intratumoral and peritumoral radiomics models demonstrated promising performance in predicting occult LNM, with the peritumoral model using 1 mm expansion showing particular efficacy. Some studies suggest 3 mm as the optimal peritumoral distance for cancer research [14, 34]. Considering that HNSCC does not have as large a tumor and peritumoral region as hepatocellular carcinoma or glioma, an excessively large peritumoral region may contain excessive normal tissue. Our preliminary comparative analysis revealed superior predictive performance at 1 mm (vs. 2–3 mm), suggesting this distance optimally captures microenvironmental features relevant to occult LNM development in HNSCC while minimizing extraneous tissue interference [35, 36]. The RF classifier outperformed both LR and SVM classifiers in predicting occult LNM. As an ensemble model, RF classifier improves model stability and robustness by constructing multiple decision trees and combining their outcomes through voting or averaging, especially for high-dimensional and complex datasets like radiomics [33, 37]. In contrast, SVM classifier is more dependent on parameter selection and kernel functions, and an increase in model complexity can lead to overfitting [38]. While LR classifier is simple and interpretable, it struggles with modeling nonlinear relationships [39]. Moreover, our findings displayed that maximum tumor diameter and histological grade were significant predictors for occult LNM, in line with findings from other studies [40, 41]. However, when further integrated into the hybrid model, they provided no additional improvement in model performance.

In the tumor microenvironment, CD8 + T cells serve as key effector cells in antitumor immune responses, eliminating tumor cells through mechanisms such as the perforin/granzyme pathway, Fas/FasL pathway. However, as the tumor microenvironment undergoes dynamic changes, CD8 + T cells may transition into an exhausted phenotype under persistent stimulation from inflammatory or immunosuppressive factors, leading to impaired immune surveillance and fostering early tumor metastasis and progression. Studies have shown that exhaustion-associated CD8 + T cells typically exhibit a range of functional impairments, including reduced cytotoxicity, diminished proliferative capacity, decreased ability to secrete cytokines, and sustained high expression of immune checkpoint molecules [42, 43]. In this study, we conducted bulk and single-cell RNA sequencing analyses to further investigate the underlying biological associations of the RF-habitat radiomics model. The results revealed that patients in the high-risk group exhibited an immunosuppressive microenvironment, primarily characterized by exhaustion-associated CD8_CXCL13 cells, which may represent the potential biological basis for the predictive capability of the RF-habitat radiomics model. Notably, the accumulation of exhaustion-associated CD8 + T cells in high-risk tumors results from two mechanisms: tumor microenvironment-induced T cell exhaustion or recruitment of immunosuppressive cells. Strikingly, radiomic features—including intensity, texture, and wavelet-derived parameters—can effectively captured these immune cell activity patterns [44, 45], providing crucial evidence for understanding the molecular mechanisms related to occult LNM. Furthermore, these findings open new research avenues for developing novel immunotherapeutic strategies for patients with HNSCC.

This study has some limitations. Firstly, one key concern was that its retrospective nature may inherently result in biases. Therefore, a multicenter prospective study is crucial for verifying the applicability of our models. Secondly, validation of the correlation between habitat subregions and pathological results was needed. However, due to the study’s retrospective design, such correlations are technically challenging to achieve. Finally, the sample size of genomic set used in this study was limited. A larger sample size in genomic analysis may be more convincing.

## Conclusion

The RF-habitat radiomics model built by venous-phase CT images demonstrated excellent performance in predicting occult LNM in HNSCC patients. Notably, biological analysis showed the RF-habitat radiomics model was connected to the infiltration of exhaustion-related CD8 + T cells, a finding that may uncover tumor microenvironment characteristics contributing to occult LNM. Further experimental validation in cellular or animal studies is needed to test the biological relevance of our genomic findings.

## Electronic supplementary material

Below is the link to the electronic supplementary material.

Supplementary Material 1

## Data Availability

The datasets used and/or analyzed during the current study are available from the corresponding author on reasonable request.

## Abbreviations

AUC: Area under the curve
cN0: Clinically LN negative
CI: Confidence interval
CNV: Copy number variation
DB: Davis-Bouldin
DEGs: Differentially expressed genes
DFS: Disease-free survival
GO: Gene ontology
GSEA: Gene set enrichment analysis
GSVA: Gene set variation analysis
HNSCC: Head and neck squamous cell carcinoma
ICCs: Intra- and interclass correlation coefficients
KEGG: Kyoto encyclopedia of genes and genomes
LASSO: Least absolute shrinkage and selection operator
LR: Logistic regression
LSCC: Laryngeal squamous cell carcinoma
LNM: Lymph node metastasis
mIF: Multiplex immunofluorescence
OR: Odds ratio
OS: Overall survival
pN-: Pathological LN negative
pN +: Pathological LN positive
RF: Random forest
ROC: Receiver operating characteristic
ROI: Region of interest
SHAP: SHapley Additive exPlanations
SVM: Support vector machine
TCIA: Cancer imaging archive
TCGA: Cancer genome atlas
UMAP: Uniform manifold approximation and projection
VOI: Volumes of interest
