# Supplementary material for: Habitat radiomics predicts occult lymph node metastasis and uncovers immune microenvironment of head and neck cancer
Source: J Transl Med. 2025 May 1;23:498. doi: 10.1186/s12967-025-06474-7 (PMC12046905; doi:10.1186/s12967-025-06474-7)
Supplement: Supplementary file 1 — Supplementary Material 1 [file 12967_2025_6474_MOESM1_ESM.docx]

**Supplemental material**

**Appendix S1**

**Radiotranscriptomic Analysis**

Based on the cutoff value (0.270) of the RF-habitat radiomics model score, patients in the genomics set were stratified into low- or high-risk groups. DGEs between the two groups were identified using the DESeq2 package with a threshold of |log2 fold change| > 1 and a *P* value < 0.05. KEGG pathway enrichment analysis was performed using the R package clusterProfiler to identify significantly enriched pathways associated with the DGEs, with a *P* value < 0.05 and a q-value < 0.05. The Benjamini-Hochberg (BH) method [1] was used for *P* value adjustment, and gene sets were restricted to sizes between 10 and 500 to ensure adequate inclusion for pathway enrichment analysis. Similarly, GSEA was conducted based on the Gene Ontology (GO) database to explore the biological processes and molecular functions that are enriched in the low- and high-risk groups.

Single-cell RNA sequencing data (10× Genomics) were processed using the R package Seurat v5.0. In order to exclude doublets and low-quality cells, the data underwent rigorous quality control to remove cells that did not meet the criteria, including those with more than 20% mitochondrial gene expression, RNA counts exceeding 40,000, or more than 6,000 gene features. Next, the R package Harmony was used to correct batch effects, eliminating batch differences between samples and ensuring the reliability and consistency of subsequent analyses. During data preprocessing, normalization, identification of highly variable genes, data scaling, and Principal Component Analysis (PCA) dimensionality reduction were performed. Subsequently, the DEGs of each cluster were identified by the “FindAllMarkers” function in Seurat, and the clusters were annotated into distinct cell types according to the expression of classic markers. Canonical markers used for cell annotation are derived from the research by HU et al. [2] Nonlinear uniform manifold approximation and projection (UMAP) was applied to visualize the data. The differences in cell proportions between the high-risk and low-risk groups were compared, and subclustering and re-annotation were performed on the subpopulations with significant differences. This allowed for the refinement of cellular composition and the identification of DGEs presented in the distinct subgroups. Additionally, gene set variation analysis (GSVA) was performed on the 50 Hallmark pathways to evaluate the pathway enrichment scores and identify the molecular characteristics associated with distinct subgroups.

To validate the differences in tumor immune microenvironment between the low-and high- risk groups, multiplex immunofluorescence (mIF) staining was employed to analyze the infiltration of CD8+ T cells in both groups of patients. By staining with CD8 (CD8+ T cell marker) and CXCL13 (exhausted T cell marker), the technique enabled the visualization of the distribution of exhausted-related CD8+ T cells in the tumor microenvironment, providing valuable insights into the immune landscape associated with different risk profiles.

Paraffin-embedded tumor tissue sections were prepared and fixed in 4% PFA. The tissues were then sectioned into 4-µm slices. Immunofluorescent staining was performed using CD8 (AiFang Biological, AF300538) and CXCL13 (AiFang Biological, AF06650) antibodies to highlight specific markers within the tissue. Images were captured with a Nikon ECLIPSE C1 microscope equipped with a high-resolution camera.

**Follow-up Strategy**

All patients underwent regular postoperative follow-up through telephone interviews, medical records, and death certificates. Follow-ups were conducted every three months during the first year after surgery, every six months for the next four years, and annually thereafter. The primary endpoints of this study were overall survival (OS) and disease-free survival (DFS). DFS was defined as the interval between the date of surgery and the occurrence of events such as disease progression, the date of the last follow-up, or death from any cause. Disease progression, including local recurrence and distant metastasis, was confirmed through clinical examination, imaging findings, or biopsy.

**Reference**

1.Benjamini Y, Hochberg Y. Controlling the false discovery rate: a practical and powerful approach to multiple testing. J Royal Statistical Soc Series B (Methodological). 1995; 57(1):289–300.

2.Hu C, Li T, Xu Y, Zhang X, Li F, Bai J, et al. Cell Marker 2.0: an updated data base of manually curated cell markers in human/mouse and web tools based on scRNA-seq data. Nucleic Acids Res. 2023;51(D1):D870–6.

**Appendix S2**

**1. Table S**

**Table S1** CT protocols of the three Centers

| **Parameters** | **Center1** |  |  | **Center2** |  | **Center3** |  |
| --- | --- | --- | --- | --- | --- | --- | --- |
| CT scanners | GE  Discovery  CT750 HD | SIEMENS SOMATOM  Definition Flash | SIEMENS SOMATOM Force | PHILIPS Brilliance iCT | PHILIPS  Brilliance CT 64  Channel | SIEMENS SOMATOM  Definition Flash | SIEMENS SOMATOM Force |
| Tube voltage | 100–120 kV | 100–120 kV | 100 kV | 100–140 kV | 100–120 kV | 100–150 kV | 120 kV |
| Gantry rotation time | 0.6 s | 0.5 s | 0.28 s | 0.27 s | 0.5 s | 0.5 s | 0.25 s |
| Detector collimation | 64×0.625 mm | 128×0.6 mm | 128×0.6 mm | 256×0.625 mm | 64×0.625 mm | 128×0.6 mm | 128×0.6 mm |
| section thickness | 5 mm | 5 mm | 5 mm | 5mm | 5 mm | 5 mm | 5 mm |
| section interval | 5 mm | 5 mm | 5 mm | 5mm | 5mm | 5 mm | 5 mm |
| Image matrix | 512×512 | 512×512 | 512×512 | 512×512 | 512×512 | 512×512 | 512×512 |
| Contrast agent type | Ioversol | Ioversol | Ioversol | Iohexol | Iohexol | Ioversol | Ioversol |
| Contrast agent concentration | 320mgI/mL | 320mgI/mL | 320mgI/mL | 350mgI/mL | 350mgI/mL | 320mgI/mL | 300mgI/mL |
| Contrast agent dosage | 1.5–2.0mL/kg | 1.5mL/kg | 1.5mL/kg | 1.5–2.0mL/kg | 1.5–2mL/kg | 1.5–2mL/kg | 1.5–2mL/kg |
| Contrast agent infused rate | 3.0–4.0mL/s | 3.0–4.0mL/s | 3.0–4.0mL/s | 3.0–4.0mL/s | 3.0–4.0mL/s | 3.0–4.0mL/s | 3.0–4.0mL/s |

Abbreviations: kV, kilovolt; mA, milliampere; mm, milimetre; mL, milliliter; kg, kilogram. Center 1, The First Affiliated Hospital of Chongqing Medical University; Center 2, The People’s Hospital of Hechuan Chongqing; Center 3, Zigong Fourth People’s Hospital

**Table S2** Nineteen radiomics features and their definitions

| **Radiomics features** | **Definition** |
| --- | --- |
| **First order** |  |
| Entropy | Entropy specifies the uncertainty/randomness in the image values. It measures the average amount of information required to encode the image values. |
| Median | Median is the median gray level intensity within the ROI |
| Mean Absolute Deviation | Mean Absolute Deviation is the mean distance of all intensity values from the Mean Value of the image array |
| Gray level co-occurrence matrix (GLCM) |  |
| Difference Average | Difference Average measures the relationship between occurrences of pairs with similar intensity values and occurrences of pairs with differing intensity values. |
| Difference Entropy | Difference Entropy is a measure of the randomness/variability in neighborhood intensity value differences. |
| Difference Variance | Difference Variance is a measure of heterogeneity that places higher weights on differing intensity level pairs that deviate more from the mean. |
| Joint Energy | Energy is a measure of homogeneous patterns in the image. A greater Energy implies that there are more instances of intensity value pairs in the image that neighbor each other at higher frequencies. |
| Joint Entropy | Joint entropy is a measure of the randomness/variability in neighborhood intensity values. |
| Inverse Variance | Inverse Variance is a measure of image homogeneity. |
| Sum Entropy | Sum Entropy is a sum of neighborhood intensity value differences. |
| IMC1 | IMC1 assesses the correlation between the probability distributions of i and j (quantifying the complexity of the texture). |
| IMC2 | IMC2 also assesses the correlation between the probability distributions of i and j (quantifying the complexity of the texture). |
| Gray level run length matrix (GLRLM) |  |
| Run Entropy | Run Entropy measures the uncertainty/randomness in the distribution of run lengths and gray levels. A higher value indicates more heterogeneity in the texture patterns. |
| Run Variance | Run Variance is a measure of the variance in runs for the run lengths. |
| Long Run Emphasis | Long Run Emphasis is a measure of the distribution of long run lengths, with a greater value indicative of longer run lengths and more coarse structural textures. |
| Size-Zone Non-Uniformity Normalized | Size-Zone Non-Uniformity Normalized measures the variability of size zone volumes throughout the image, with a lower value indicating more homogeneity among zone size volumes in the image. |
| Small Area High Gray Level Emphasis | Small Area High Gray Level Emphasis measures the proportion in the image of the joint distribution of smaller size zones with higher gray-level values. |
| Neighbouring gray tone difference matrix (NGTDM) |  |
| Contrast | Contrast is a measure of the spatial intensity change, but is also dependent on the overall gray level dynamic range. Contrast is high when both the dynamic range and the spatial change rate are high. |
| Strength | Strength is a measure of the primitives in an image. Its value is high when the primitives are easily defined and visible. |

**Table S3** Performance comparison of different radiomics models for predicting occult LNM.

| **Cohort** | **Model** | **AUC (95%CI)** | **Accuracy** | **Sensitivity** | **Specificity** | **PPV** | **NPV** |
| --- | --- | --- | --- | --- | --- | --- | --- |
| Training set | SVM-habitat radiomics model | 0.942 [0.906–0.978] | 0.897 | 0.942 | 0.881 | 0.736 | 0.977 |
|  | RF-habitat radiomics model | 0.919 [0.886–0.951] | 0.842 | 0.860 | 0.836 | 0.649 | 0.944 |
|  | LR-habitat radiomics model | 0.910 [0.874–0.945] | 0.879 | 0.756 | 0.922 | 0.774 | 0.915 |
|  | SVM-peri-radiomics model | 0.914 [0.876–0.951] | 0.848 | 0.919 | 0.824 | 0.648 | 0.966 |
|  | RF-peri-radiomics model | 0.874 [0.830–0.917] | 0.797 | 0.791 | 0.799 | 0.581 | 0.915 |
|  | LR-peri-radiomics model | 0.865 [0.822–0.907] | 0.773 | 0.767 | 0.775 | 0.545 | 0.904 |
|  | SVM-intra-radiomics model | 0.927 [0.891–0.962] | 0.842 | 0.930 | 0.811 | 0.635 | 0.971 |
|  | RF-intra-radiomics model | 0.871 [0.826–0.915] | 0.836 | 0.674 | 0.893 | 0.690 | 0.886 |
|  | LR-intra-radiomics model | 0.885 [0.844–0.925] | 0.809 | 0.791 | 0.816 | 0.602 | 0.917 |
| Internal test set | SVM-habitat radiomics model | 0.764 [0.675–0.852] | 0.786 | 0.625 | 0.858 | 0.667 | 0.835 |
|  | RF-habitat radiomics model | 0.857 [0.780–0.933] | 0.864 | 0.750 | 0.915 | 0.800 | 0.890 |
|  | LR-habitat radiomics model | 0.706 [0.615–0.796] | 0.597 | 0.812 | 0.500 | 0.424 | 0.855 |
|  | SVM-peri-radiomics model | 0.812 [0.741–0.883] | 0.727 | 0.729 | 0.726 | 0.547 | 0.856 |
|  | RF-peri-radiomics model | 0.851 [0.783–0.918] | 0.786 | 0.729 | 0.811 | 0.636 | 0.869 |
|  | LR-peri-radiomics model | 0.774 [0.691–0.857] | 0.773 | 0.604 | 0.849 | 0.644 | 0.826 |
|  | SVM-intra-radiomics model | 0.796 [0.709–0.881] | 0.805 | 0.646 | 0.877 | 0.705 | 0.845 |
|  | RF-intra-radiomics model | 0.825 [0.751–0.899] | 0.760 | 0.812 | 0.736 | 0.582 | 0.897 |
|  | LR-intra-radiomics model | 0.782 [0.699–0.863] | 0.714 | 0.792 | 0.679 | 0.528 | 0.878 |
| External test set | SVM-habitat radiomics model | 0.763 [0.685–0.841] | 0.787 | 0.475 | 0.943 | 0.806 | 0.782 |
|  | RF-habitat radiomics model | 0.835 [0.765–0.904] | 0.836 | 0.639 | 0.934 | 0.830 | 0.838 |
|  | LR-habitat radiomics model | 0.736 [0.652–0.819] | 0.760 | 0.574 | 0.852 | 0.660 | 0.800 |
|  | SVM-peri-radiomics model | 0.782 [0.790–0.944] | 0.803 | 0.541 | 0.934 | 0.805 | 0.803 |
|  | RF-peri-radiomics model | 0.790 [0.720–0.859] | 0.787 | 0.443 | 0.959 | 0.844 | 0.775 |
|  | LR-peri-radiomics model | 0.781 [0.709–0.851] | 0.721 | 0.770 | 0.697 | 0.560 | 0.859 |
|  | SVM-intra-radiomics model | 0.785 [0.713–0.856] | 0.776 | 0.590 | 0.869 | 0.692 | 0.809 |
|  | RF-intra-radiomics model | 0.827 [0.766–0.887] | 0.787 | 0.754 | 0.803 | 0.657 | 0.867 |
|  | LR-intra-radiomics model | 0.748 [0.674–0.822] | 0.732 | 0.639 | 0.779 | 0.591 | 0.812 |

Abbreviations: LR, Logistic Regression; SVM, Support Vector Machine; RF, Random Forest; AUC, area under the curve; CI, confidence interval; PPV, positive predictive value; NPV, negative predictive value

**Table S4** Clinical characteristics of eligible patients with HNSCC for prognostic analysis.

| **Clinical characteristics** | **Training set (n = 208)** | **Internal test set (n = 107)** | **External test set (n = 123)** |
| --- | --- | --- | --- |
| Age (mean ± SD, years) | 62.27±8.37 | 61.90±8.80 | 61.81±9.30 |
| Sex |  |  |  |
| Male | 196(94.23) | 103(96.26) | 106(86.18) |
| Female | 12(5.77) | 4(3.74) | 17(13.82) |
| Somking |  |  |  |
| Yes | 193(92.79) | 98(91.59) | 97(78.86) |
| No | 15(7.21) | 9(8.41) | 26(21.14) |
| Alcohol consumption |  |  |  |
| Yes | 138(66.35) | 75(70.09) | 75(60.98) |
| No | 70(33.65) | 32(29.91) | 48(39.02) |
| Histological grade |  |  |  |
| Poor | 36(17.31) | 11(10.28) | 13(10.57) |
| Moderate | 98(47.12) | 50(46.73) | 56(45.53) |
| Well | 74(35.58) | 46(42.99) | 54(43.90) |
| Clinic T stage |  |  |  |
| T1 | 56(26.92) | 20(18.69) | 39(31.71) |
| T2 | 67(32.21) | 23(21.50) | 23(18.70) |
| T3 | 56(26.92) | 52(48.60) | 48(39.02) |
| T4 | 29(13.94) | 12(11.21) | 13(10.57) |
| Radiologic features-tumor volume (cm3) | 7.49±6.21 | 9.24±12.95 | 8.72±7.74 |
| Radiologic features-maximum tumor diameter (cm) | 2.98±1.16 | 2.93±1.44 | 3.16±1.17 |
| Radiologic features-enhancement patterns |  |  |  |
| Homogenous enhancement | 150(72.12) | 78(72.90) | 82(66.67) |
| Heterogeneous enhancement | 58(27.88) | 29(27.10) | 41(33.33) |
| Radiologic features-intratumoral necrosis |  |  |  |
| Yes | 28(13.46) | 13(12.15) | 19(15.45) |
| No | 180(86.54) | 94(87.85) | 104(84.55) |
| RF-habitat radiomics score | 0.27±0.25 | 0.23±0.21 | 0.25±0.17 |
| Follow-up time (months) |  |  |  |
| Median† | 34 (16–66) | 25.5(14–45.5) | 34 (16–50) |
| Maximum | 113 | 119 | 93 |

Abbreviations: RF, Random Forest; SD, standard deviation.

†Data in parentheses are interquartile ranges.

**2 Figure S**

**
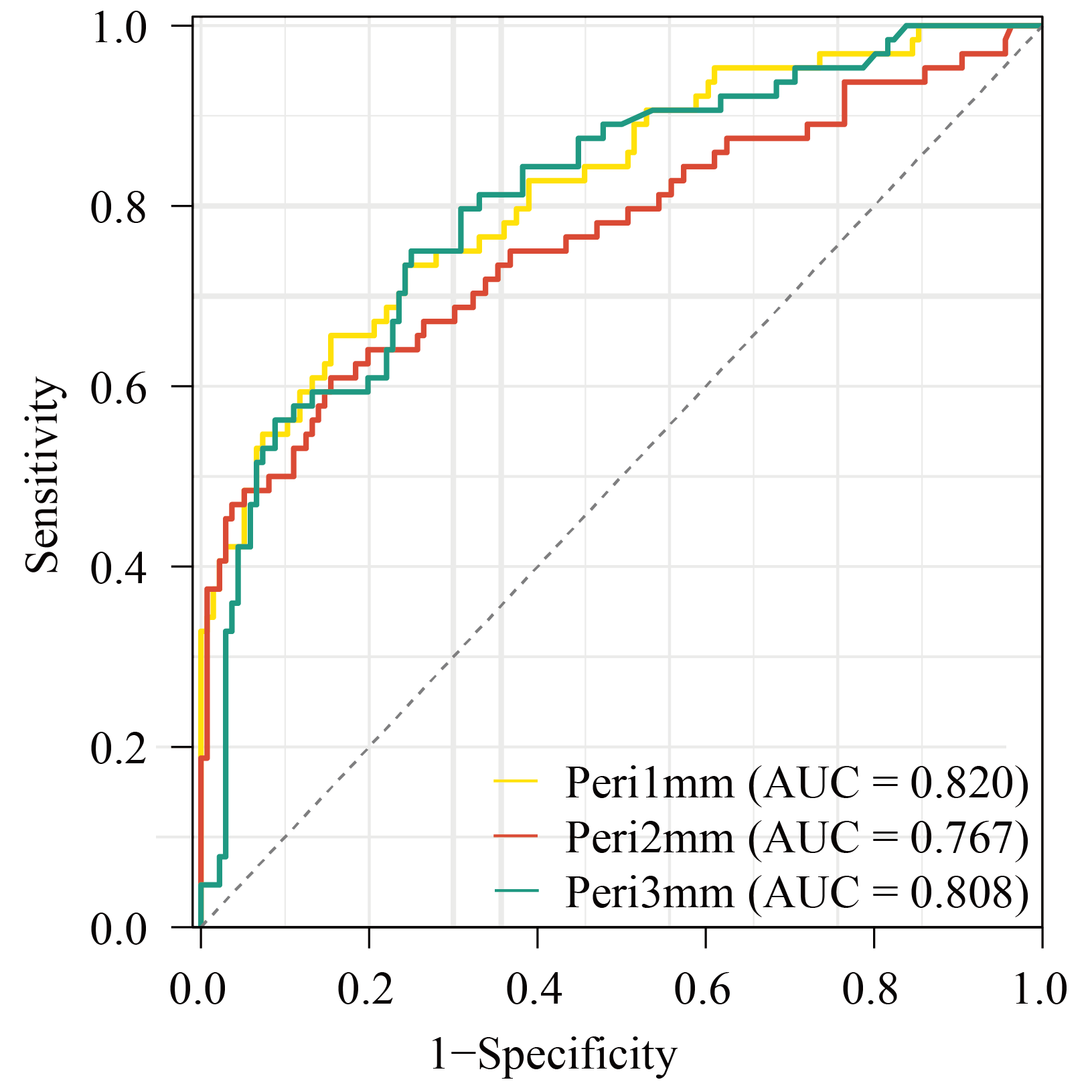
**

**Figure S1** Preliminary experiment (200 patients) on model performance with different expansion lengths. The peritumoral radiomics model with a 1mm expansion length achieved the best performance, with an AUC of 0.820. Subsequent studies will be based on the 1mm expansion

**
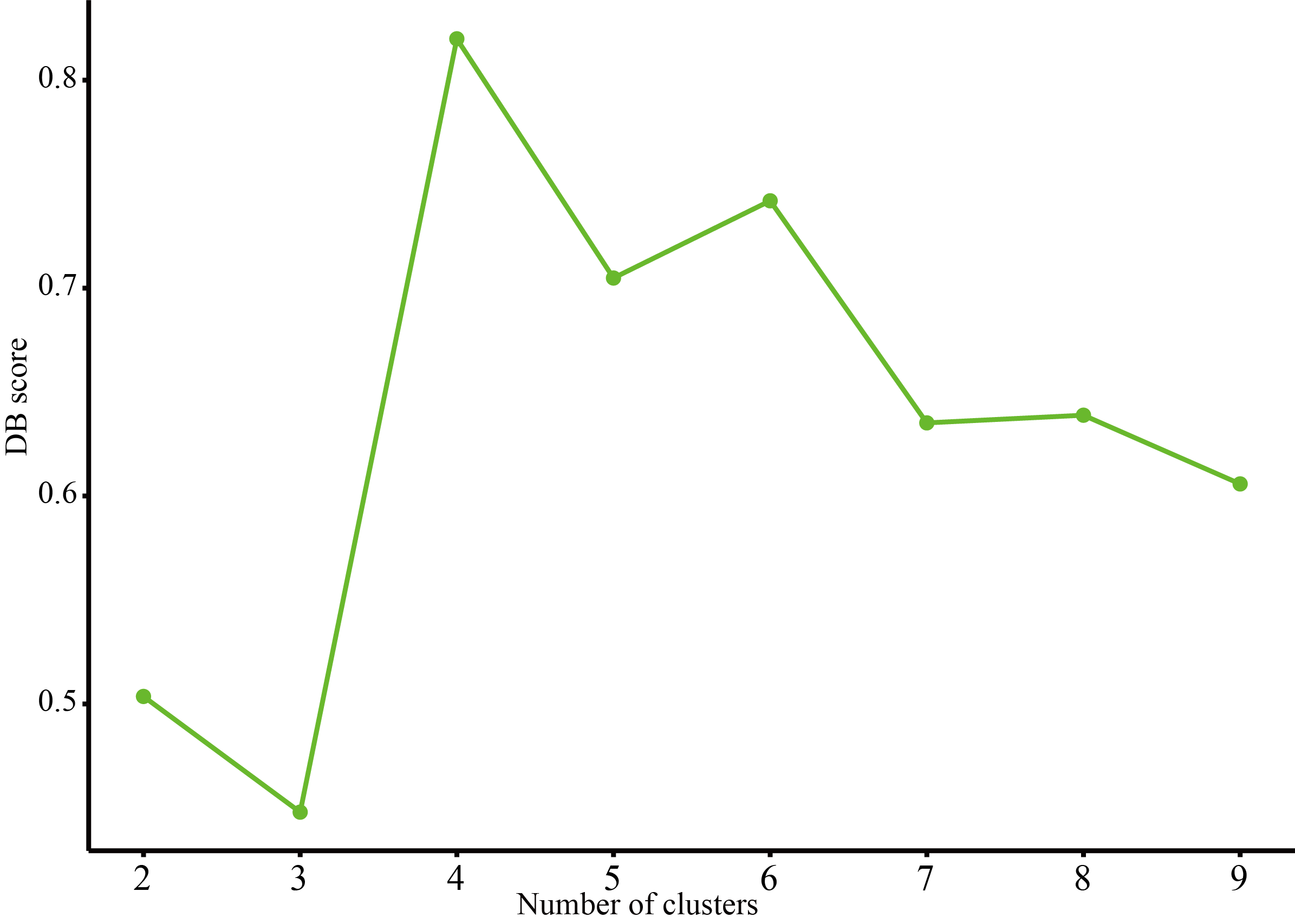
**

**Figure S2** Evaluation of Clustering Quality Using Davies-Bouldin (DB) Score for Different Numbers of Clusters. The DB score was a metric used to evaluate clustering quality, with lower scores indicating better clustering performance. The data points and line indicate that the model performs best with 3 clusters, where the DB score was the lowest

**
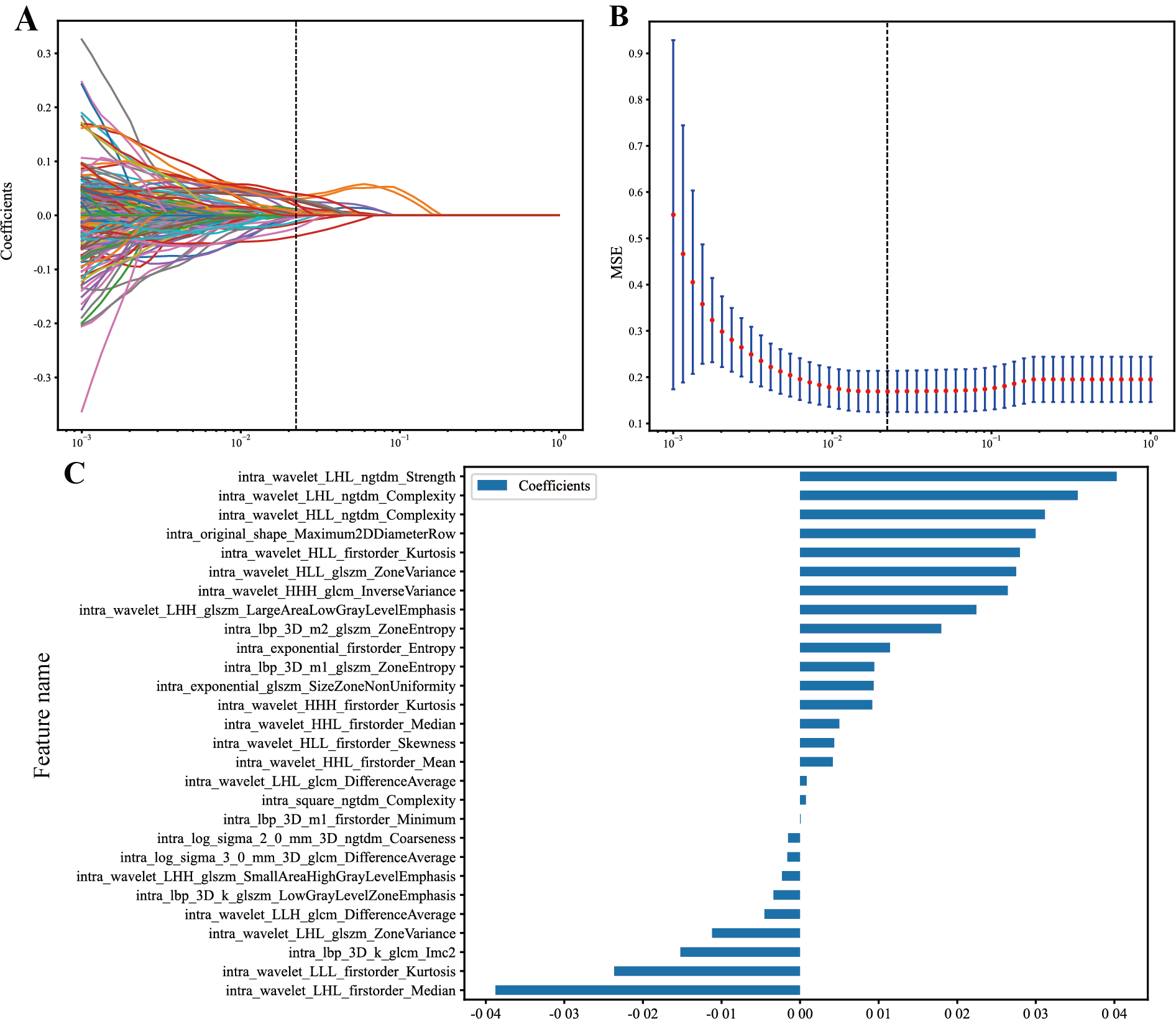
**

**Figure S3** The results of LASSO regression for intratumoral radiomics features selection. Feature coefficient trajectories (A) and the mean squared error (MSE) curve from 10-fold cross-validation (B) were shown across different λ values. The dashed line indicated the optimal λ, determined as the value minimizing the MSE. (C) A total of 28 selected radiomics features and their corresponding coefficients at the optimal λ were displayed in a bar plot, ranked by absolute values to reflect their importance in the model

**
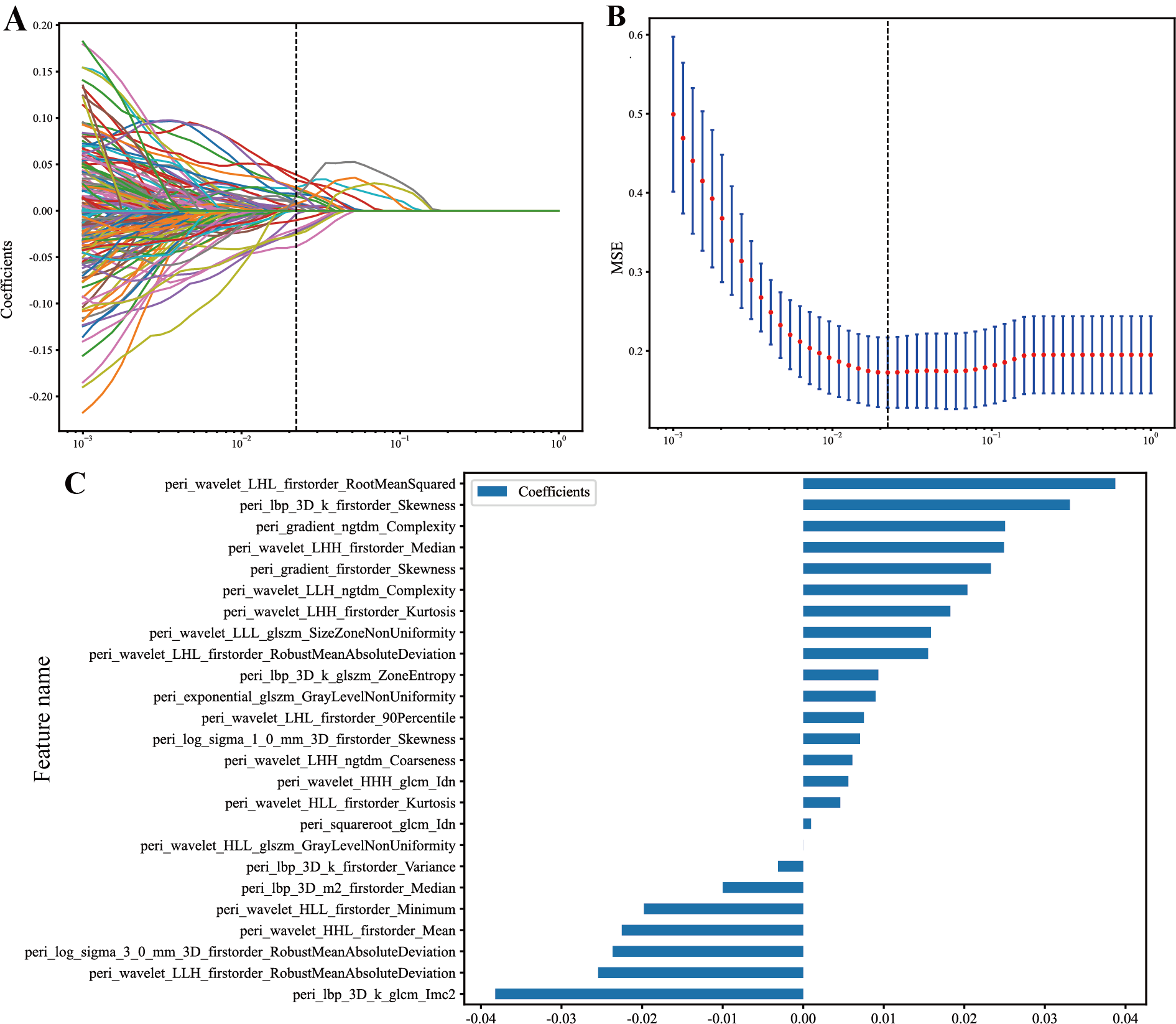
**

**Figure S4** The results of LASSO regression for peritumoral radiomics features selection. Feature coefficient trajectories (A) and the mean squared error (MSE) curve from 10-fold cross-validation (B) were shown across different λ values. The dashed line indicated the optimal λ, determined as the value minimizing the MSE. (C) A total of 25 selected radiomics features and their corresponding coefficients at the optimal λ were displayed in a bar plot, ranked by absolute values to reflect their importance in the model

**
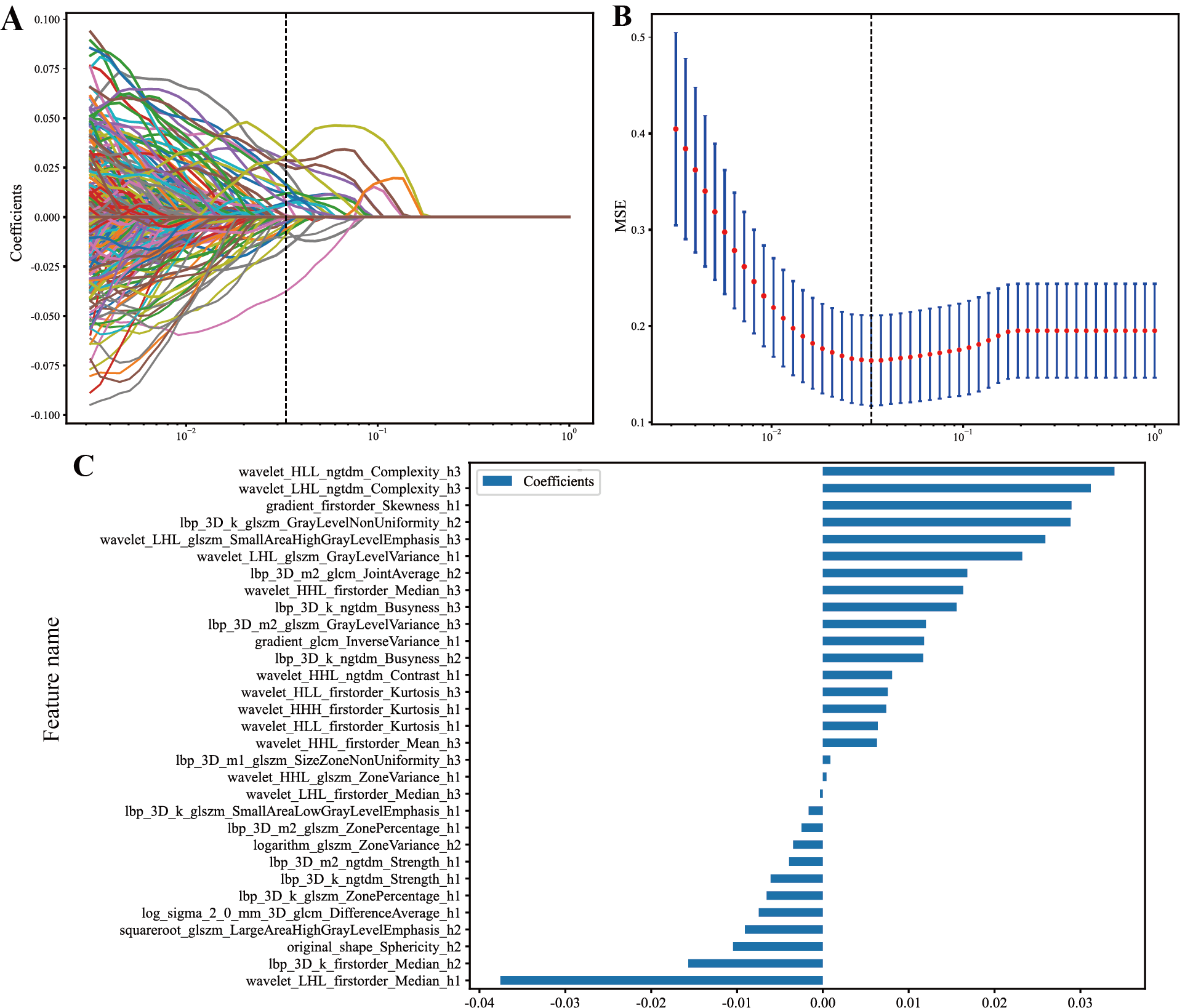
**

**Figure S5** The results of LASSO regression for habitat radiomics features selection. Feature coefficient trajectories (A) and the mean squared error (MSE) curve from 10-fold cross-validation (B) were shown across different λ values. The dashed line indicated the optimal λ, determined as the value minimizing the MSE. (C) A total of 31 selected radiomics features and their corresponding coefficients at the optimal λ were displayed in a bar plot, ranked by absolute values to reflect their importance in the model

**
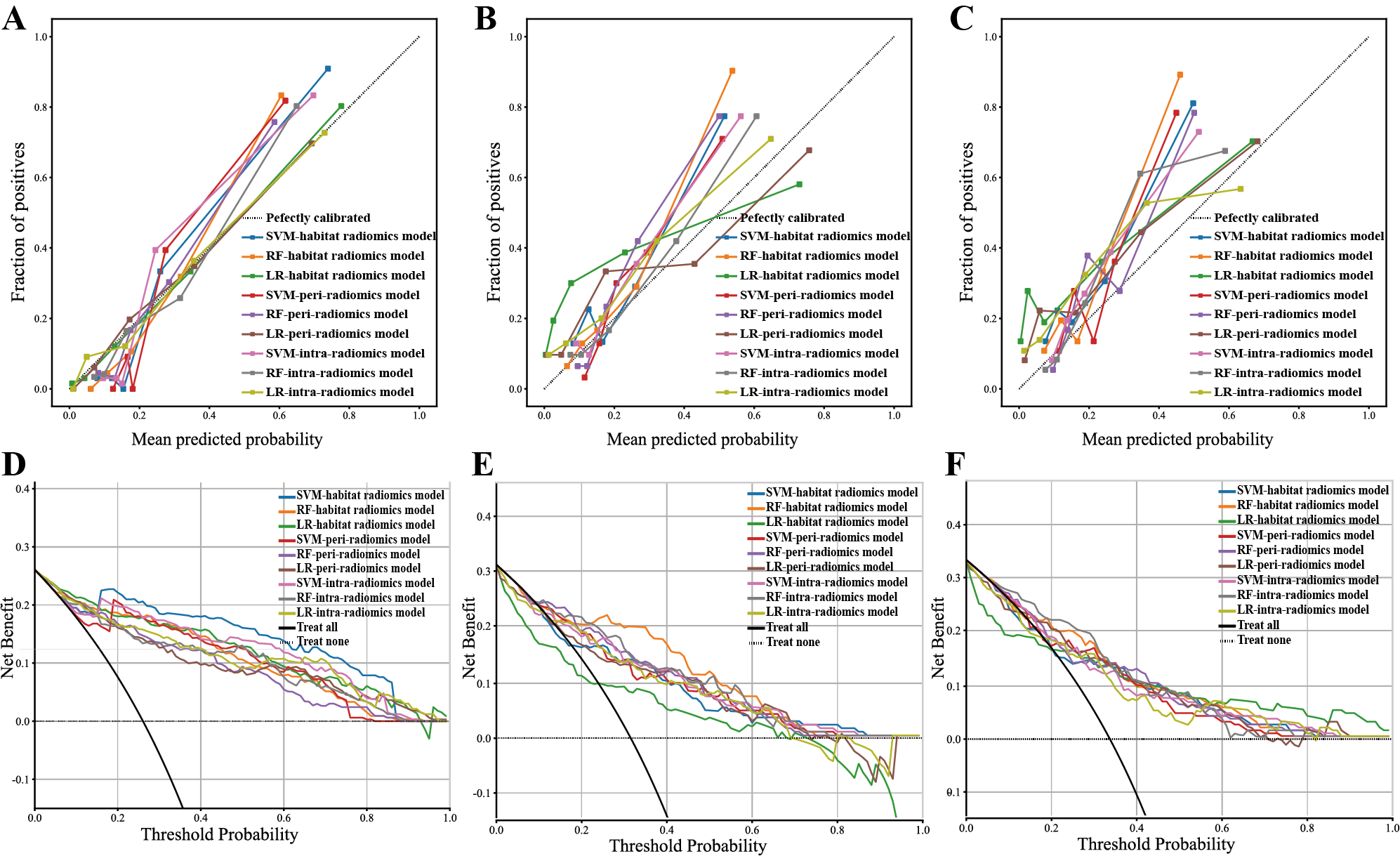
**

**Figure S6** Calibration curves of different radiomics models in the training set (A), the internal test set (B), and the external test set (C); Decision curves of different radiomics models in the training set (D), the internal test set (E), and the external test set (F)

**
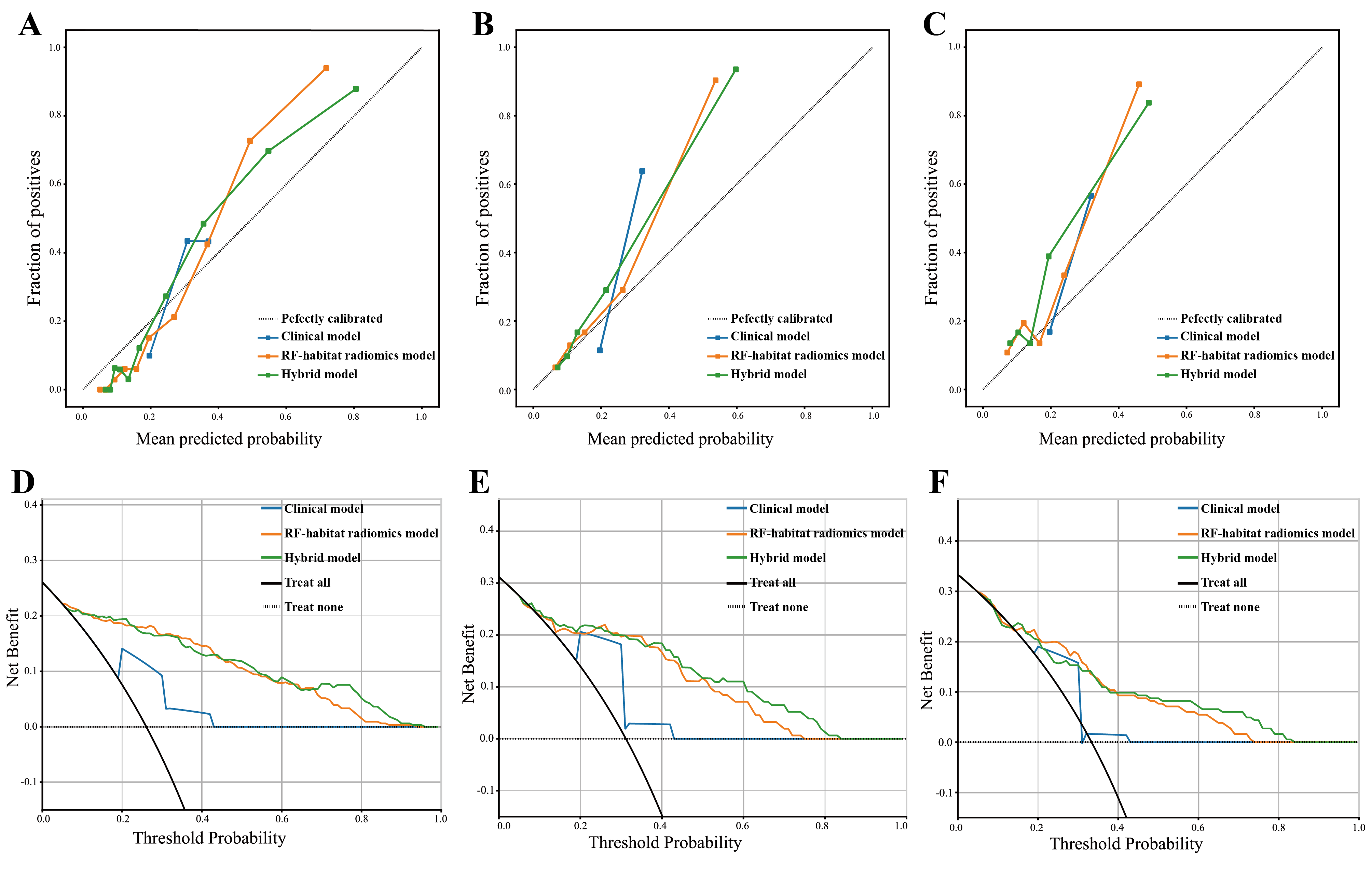
**

**Figure S7** Calibration curves of the clinical, RF-habitat radiomics, and hybrid models in the training set (A), the internal test set (B), and the external test set (C); Decision curves of the clinical, RF-habitat radiomics, and hybrid models in the training set (D), the internal test set (E), and the external test set (F)

**
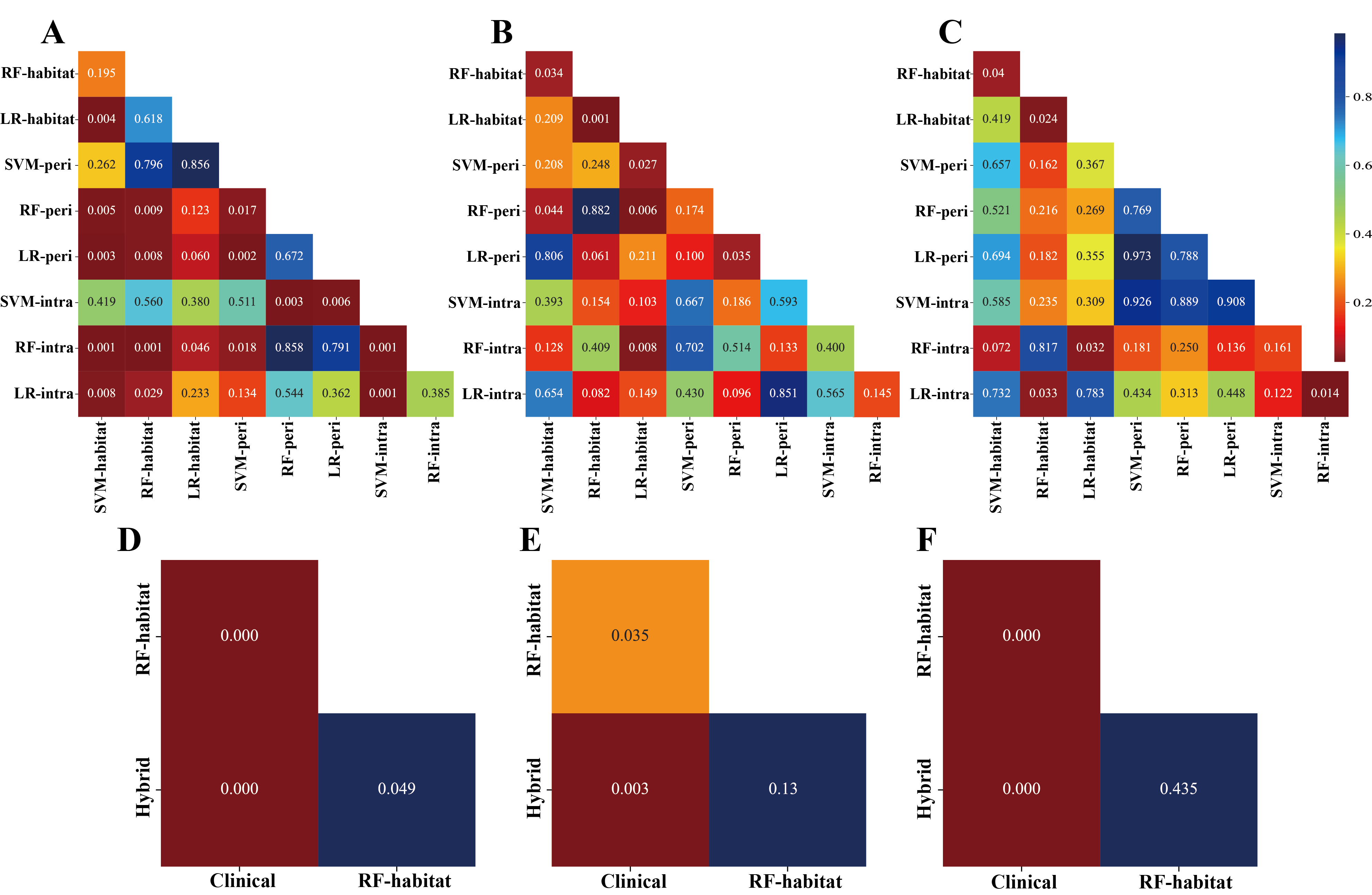
**

**Figure S8** Comparison of the performance of different models in the training set (A, D), internal test set (B, E), and the external test set (C, F), respectively. The figures in the box represent the *P* value of DeLong test

**
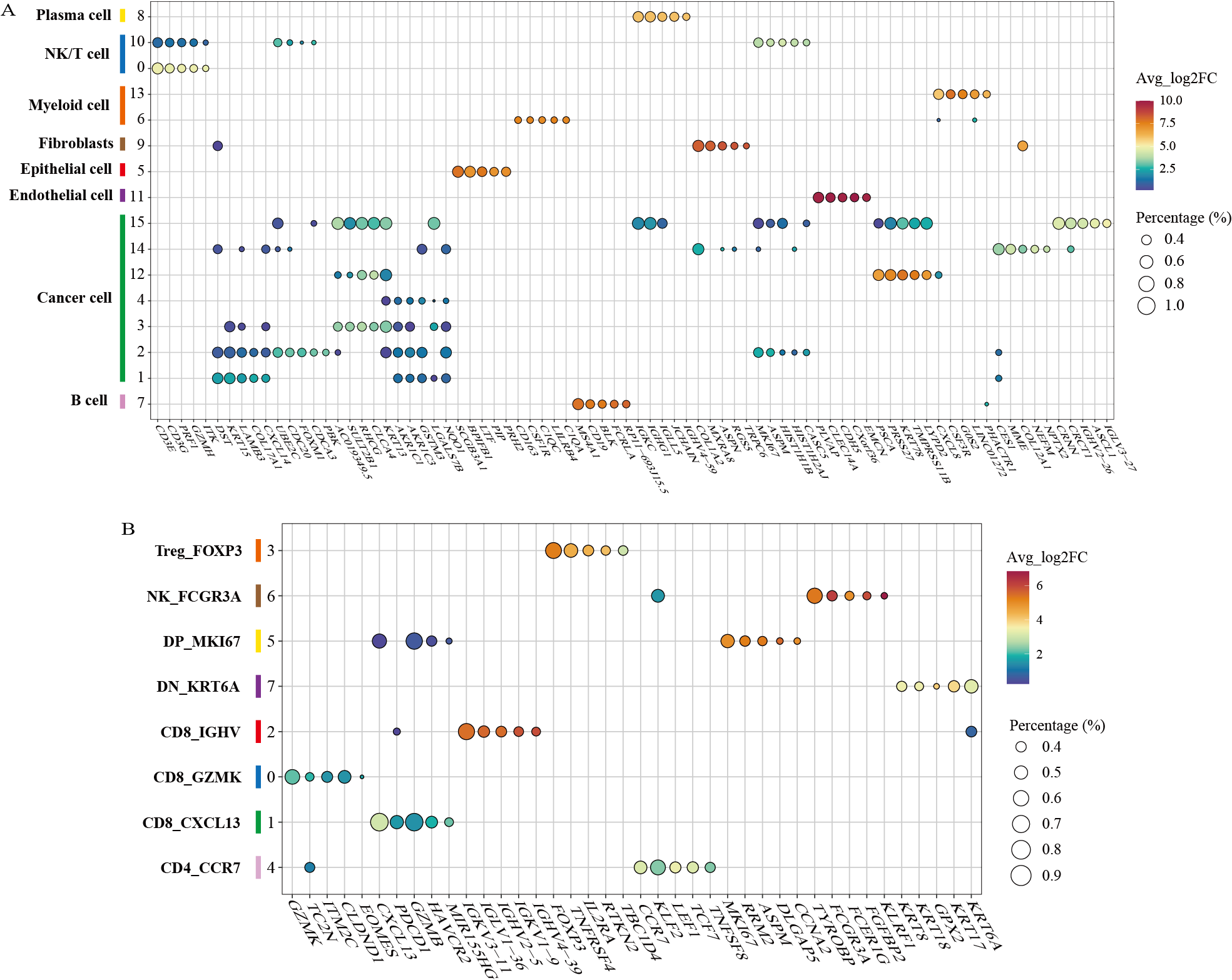
**

**Figure S9** Bubble plot of top five marker genes for all cell types (A) and NK/T subsets (B)


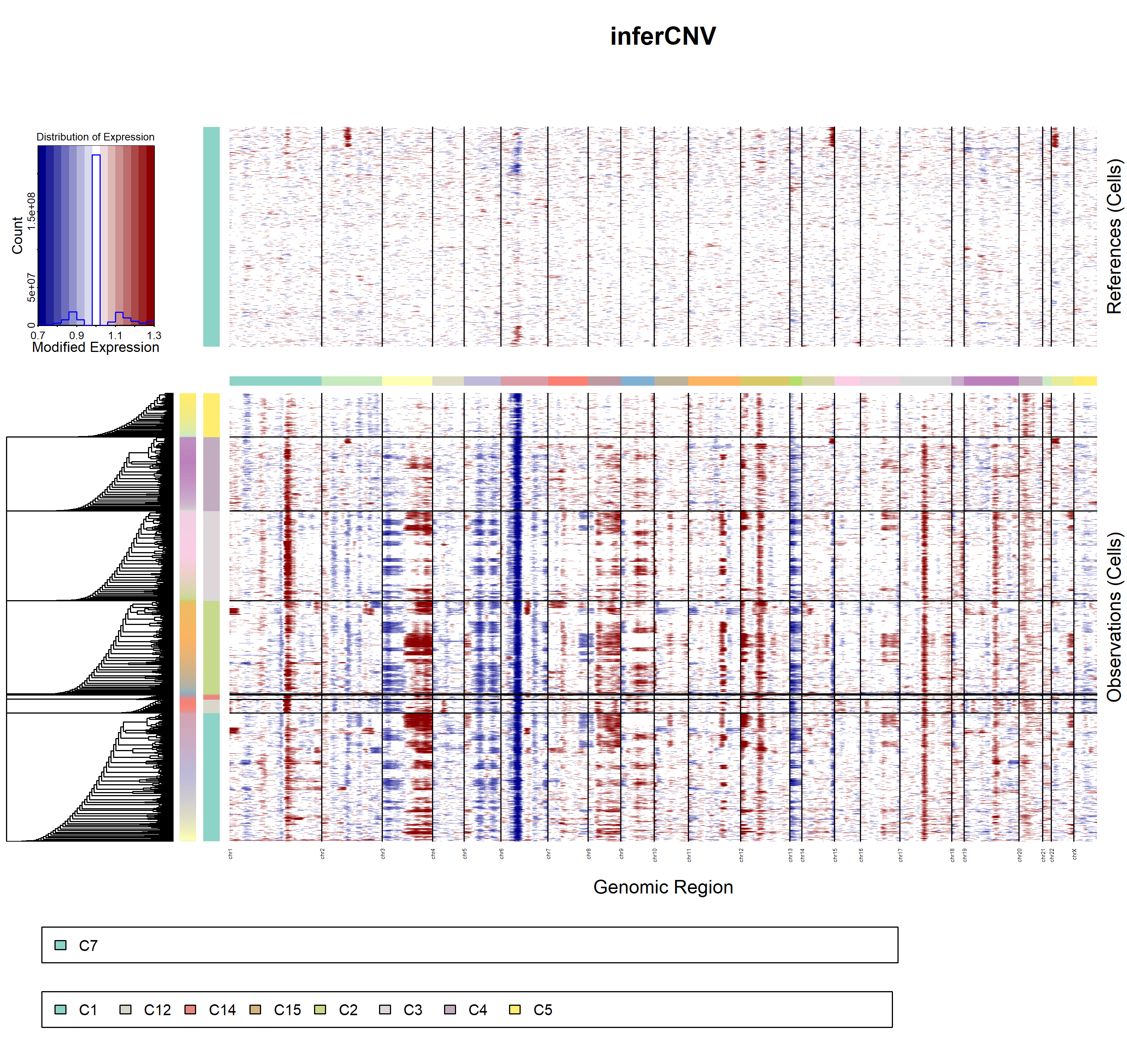


**Figure S10** InferCNV results across different clusters in epithelial cell. The heatmap displayed the modified gene expression profiles across different genomic regions, using B cell (C7) as a reference to analyze the gene expression intensity at various positions on the genome for each epithelial cell cluster (C1, C12, C14, C15, C2, C3, C4, C5). Each vertical line represented a specific genomic region, and the color intensity (from blue to red) indicated the gene copy number variation (CNV) in the chromosomal region, with red representing chromosomal region amplification and blue representing chromosomal region deletion


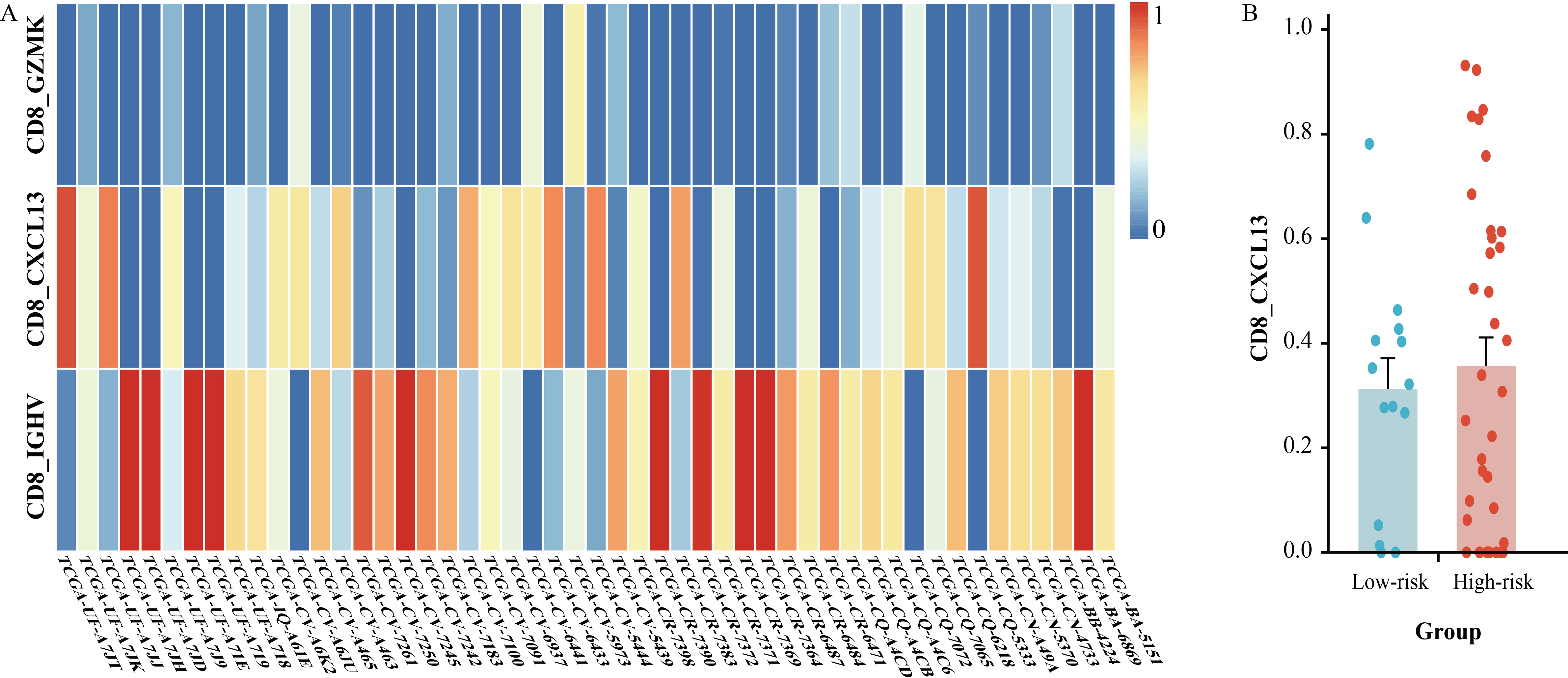


**Figure S11** CIBERSORTx deconvolution of CD8+ T cell subsets in bulk RNA data. (A) Heatmap showing the estimated proportions of CD8+ T cell subsets across 50 bulk RNA sequencing samples, obtained using the CIBERSORTx deconvolution algorithm. Each column represents an individual sample, with color intensity indicating the relative abundance of each CD8+ T cell subset. (B) Comparison of the CD8_CXCL13 cells proportions between the high- and low-risk groups. Each dot represents a sample, colored according to its risk status
